# Inhibiting the two-component system GraXRS with verteporfin to combat *Staphylococcus aureus* infections

**DOI:** 10.1038/s41598-020-74873-5

**Published:** 2020-10-21

**Authors:** Juana María Prieto, Beatriz Rapún-Araiz, Carmen Gil, José R. Penadés, Iñigo Lasa, Cristina Latasa

**Affiliations:** 1 RECOMBINA SL, Calle Nueva, 8 local 10, Mutilva 31192, Navarra, Spain; 2 Laboratory of Microbial Pathogenesis, Navarrabiomed, Complejo Hospitalario de Navarra (CHN), Universidad Pública de Navarra (UPNA), IDISNA, 31008 Pamplona, Spain; 3 Institute of Infection, Immunity and Inflammation, College of Medical, Veterinary and Life Sciences, University of Glasgow, Glasgow, G12 8TA UK

**Keywords:** Biotechnology, Microbiology, Molecular biology

## Abstract

Infections caused by *Staphylococcus aureus* pose a serious and sometimes fatal health issue. With the aim of exploring a novel therapeutic approach, we chose GraXRS, a Two-Component System (TCS) that determines bacterial resilience against host innate immune barriers, as an alternative target to disarm *S. aureus*. Following a drug repurposing methodology, and taking advantage of a singular staphylococcal strain that lacks the whole TCS machinery but the target one, we screened 1.280 off-patent FDA-approved drug for GraXRS inhibition. Reinforcing the connection between this signaling pathway and redox sensing, we found that antioxidant and redox-active molecules were capable of reducing the expression of the GraXRS regulon. Among all the compounds, verteporfin (VER) was really efficient in enhancing PMN-mediated bacterial killing, while topical administration of such drug in a murine model of surgical wound infection significantly reduced the bacterial load. Experiments relying on the chemical mimicry existing between VER and heme group suggest that redox active residue C227 of GraS participates in the inhibition exerted by this FDA-approved drug. Based on these results, we propose VER as a promising candidate for sensitizing *S. aureus* that could be helpful to combat persistent or antibiotic-resistant infections.

## Introduction

Though the undeniable efficiency of anti-infective measures like vaccines and antimicrobials have made us believe that infectious diseases are nowadays under control, nothing could be further from the truth. An increasing number of studies alert that unless actions are taken, infections caused by antibiotic-resistant bacteria will kill an extra 10 million people a year worldwide by 2050^1^.

*Staphylococcus aureus* is one of the bacterial species whose manage is especially challenging due to the emergence of methicillin-resistant (MRSA) strains. With a population-weighted mean of invasive MRSA strains of about 17% in terms of European prevalence^2^, the major health care concern related to MRSA incidence lies in the limitations of currently approved treatments, which, in turn, leads to high rates of morbidity and mortality even in industrialized nations.

Despite of being an inoffensive colonizer of the nasal epithelium of one-third of the general population^3^, *Staphylococcus aureus* might become a dangerous life-threatening pathogen when it defeats host immune system, crosses the epithelial barrier and get access to deeper tissues like blood, dermis, gastrointestinal tract, heart valves or bones^3,4^. This biological versatility is based on a highly orchestrated regulation of circuits that sense a plethora of environmental signals and modulate gene expression for fine tuning crucial traits like cell-wall structure, biofilm formation or resistance to antibiotics. The core feature of such circuits is the two-component-signaling transduction system (TCS)^5^, which is actually one of the most conserved and effective mechanisms in nature for coupling external stimuli and gene expression. In its most basic form, a canonical TCS normally consists of a membrane-bound histidine kinase and a cytosolic response regulator that, once phosphorylated, elicit appropriate changes in the cell by regulating gene expression, protein interactions, or enzymatic activity^6^.

Over the last decade, the scientific community has gained in-depth knowledge of the genes affected by specific staphylococcal TCSs, giving rise to a vast body of bibliography and information about mutants in the respective sensor kinases, response regulators and auxiliary genes. Thus, the pivotal role of AgrCA and SaeRS TCSs on virulence gene expression or the involvement of BraRS and GraXRS in antibiotic resistance has been studied in great detail^7–10^. Furthermore, since TCSs are a matter of life and death to bacteria and they are not present in host’s cells, these regulatory pathways have always been listed as promising antibacterial targets. Precisely based on the assumption that solution to present-day therapeutic limitations might somehow lie on impairing the way *Staphylococcus* senses and integrates environmental stimuli, significant effort in the form of ambitious High Throughput Screenings (HTSs) and Structure-Based Virtual Screenings (SBVSs) has been done to find new molecules with inhibitory effects on staphylococcal sensor kinases^11^. However, with few exceptions such as the molecule named walkmycin B^12^, biochemical screens normally identify a high number of compounds acting through nonspecific inhibitory mechanisms and thus render non-viable drugs in terms of clinical application^11,13,14^.

With the aim of designing a whole-cell drug discovery tool that could complement in silico docking and crystallographic analysis of kinase-ligands structure in TCS-targeting approaches, we decided to explore the potential of a recently developed staphylococcal strain that lacks the whole non-essential TCS machinery (∆XV strain)^15^. Among all the TCSs whose individual contribution to a specific *S. aureus* phenotype has been defined, and based on its overall responsibility for resistance to host defenses like polymorphonuclear cells (PMNs) or cationic antimicrobial peptides, we selected GraXRS as a candidate of therapeutic target^9,16–19^. Additional evidence supporting our choice was given by a recent work showing that *S. aureus* uses this regulatory system to sense and adapt to the acidified phagolysosome in macrophages^20^, but also by several previous studies unveiling the potential of GraXRS to impact the bacterial capacity to colonize and survive on aortic valves in a rabbit endocarditis infection model^18^ or to play a crucial function in a murine model of systemic infection^16^. Most of these preceding articles conclude that mechanisms underlying GraXRS activity are related with changes in bacterial surface charge via its target downstream gene *mpr*F and the operon *dlt*ABCD^9,17,18,21^.

In this study, we have used the *S. aureus* strain deprived of fifteen TCS^15^ and isogenic derivatives containing exclusively the GraXRS TCS as a whole-cell platform to identify drugs that specifically target this signaling pathway. Upon evaluating the GraXRS-blocking activity of 1280 FDA-approved off-patent drugs, we found that molecules with antioxidant activity as acetylsalicylic acid, ascorbic acid, the porphyrin derivative verteporfin, or the flavonoid hesperidin, are capable of inhibiting the activity of GraXRS-dependent promoters. Among all the compounds, only verteporfin made a significant contribution to the susceptibility of *S. aureus* to human PMNs-mediated killing and rendered lower levels of bacterial colonization when its effect was assessed using an in vivo murine model. Though further analysis is needed to fully understand the precise molecular targets of verteporfin, data presented in this work suggest that the redox-active cysteine of GraS is required for this molecule to exert its inhibiting effect. Altogether, our results enlighten the potential of verteporfin as a supplement and(or) alternative antimicrobial therapy and provide evidence that this compound could be included in a recently described category of drugs known as “Potential Drugs for Repurposing against Infectious Agents”.

## Results

### Design and validation of a GraXRS-focused screening platform

**Figure 1:**
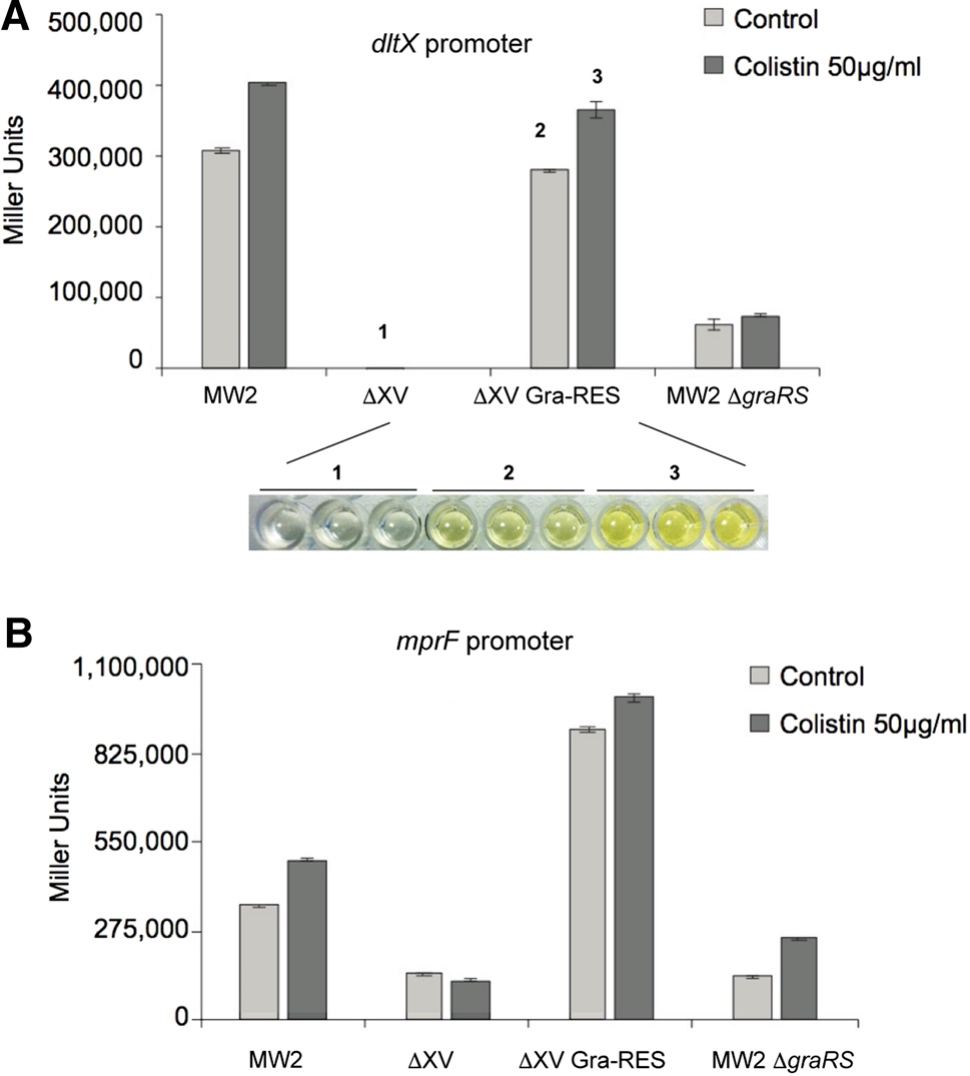
Validation of GraXRS-dependent transcriptional fusions. Graphic illustration of GraXRS-dependent activity of *dlt*X (**A**) and *mpr*F (**B**) promoters in *S. aureus* MW2 wild type, ∆XV, ∆XV Gra-RES and ∆*gra*RS strains. Transcriptional activity in TSB medium (light gray bars) and TSB supplemented with colistin 50 μg/ml (dark grey bars) is presented. Means and standard deviations values are shown from at least three independent experiments. A visual example showing different degrees of GraXRS activity [∆XV (1), ∆XV Gra-RES (2) and ∆XV Gra-RES in the presence of colistin (3)] in the 96-well format is also shown.

With the aim of designing a highly specific GraXRS-targeting screening assay, we first restored the GraXRS TCS into the chromosome of *S. aureus* ΔXV strain, which only contains the essential WalKS TCS system in its genome. The resulting *S. aureus* ΔXV Gra-RES strain, together with the corresponding *S. aureus* MW2 wild type strain and the single GraXRS mutant derivative, were transformed with two different reporter plasmids in which *lac*Z expression depends on the GraXRS-regulated promoters of *mpr*F and *dlt*X^9^. As shown in Fig. 1, transcriptional activity of reporter genes was barely detectable in the GraXRS deficient strains (single and multiple ΔXV mutants), whereas *mpr*F and *dlt*X-based reporter constructs were highly induced in the GraXRS containing strains (wild type and ΔXV GraRES). As an additional test for evaluating the behavior of the reporter strain-set, positive response of the GraXRS-dependent promoters to the presence of sublethal concentrations of colistin was also analyzed. Noticeably, and confirming the concept of TCS as self-sufficient modules previously envisioned^15^, *lac*Z expression driven by *dlt*X promoter was essentially equal in the wild type and *graXRS* restored genetic backgrounds. This result validates both the use of the GraXRS restored strain and *dltXP*::*lac*Z transcriptional fusion for high-throughput-screening designs aimed at the discovery of GraXRS-blocking drugs and molecules.

### Primary and secondary screenings

**Figure 2:**
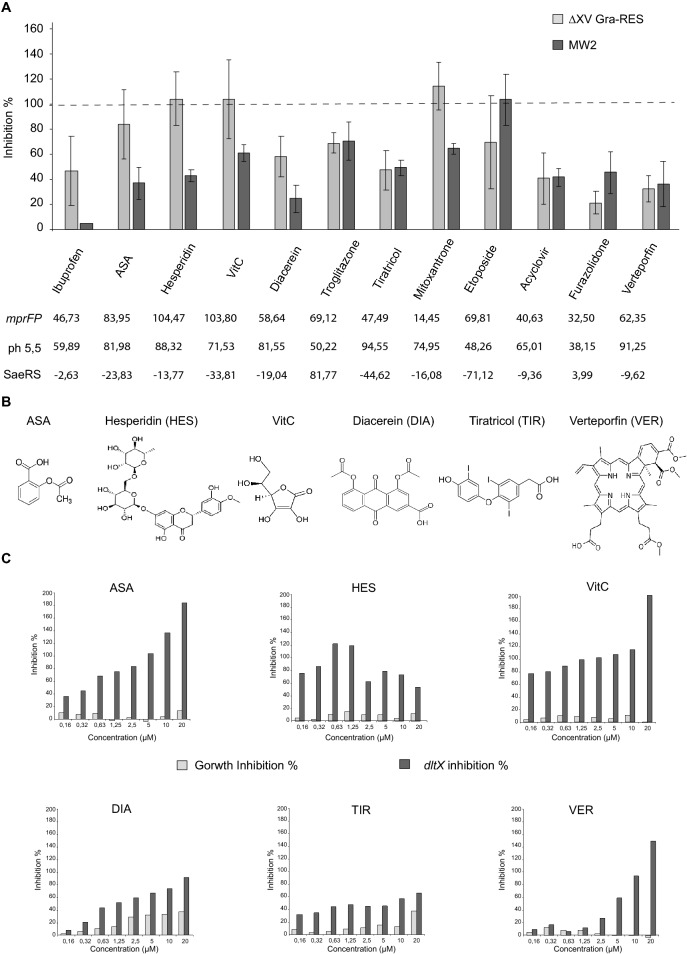
Screening results. (**A**) Graphic illustration of GraXRS-dependent activity of *dltX* promoter in the presence of selected drugs (10 μM) in *S. aureus* ∆XV Gra-RES (light gray bars) and MW2 wild type strains (dark grey bars). Data are presented as relative M.U. values obtained in the absence of any compound for *S. aureus* ∆XV Gra-RES and MW2 wild type strains respectively. Values exceeding 100% correspond to those cases where the observed transcriptional activity is lower to that showed by ∆XV and/or bacterial growth is inhibited. Standard deviations values correspond to at least three independent experiments. Results regarding additional selection criteria including the repression of the GraXRS-dependent alternative *mprF* promoter, significative inhibition of wild type strain under acidic growth conditions (pH 5.5) and transcriptional co-inhibition of the SaeRS-dependent *sec*4 promoter are presented. (**B**) Structure of selected drugs included in drug bank profiles (https://www.drugbank.ca) is shown. (**C**) Graphic illustration of Dose Dependency profiles in terms of *dlxP* inhibition exhibited by selected compounds. Data are presented as relative M.U. values obtained in the absence of any compound for *S. aureus* ∆XV Gra-RES (dark grey bars). Relative percentage of survival in the presence of increasing doses of compounds is also included (light grey bars).

From the initial screening of the 1280 drugs included in the Prestwick library (https://www.prestwickchemical.com/libraries-screening-lib-pcl.html), we selected 77 compounds that led to reduced activity of *dltX* promoter by more than 55% as determined by beta-galactosidase activity (see table in the supplemental material). Because the intention of this work was to repurpose those FDA-approved drugs that specifically targeted GraXRS-mediated signaling pathway, and this TCS has been shown to be crucial for bacterial growth under acidic conditions^15^, the capacity of the selected compounds to affect OD_600_ values at pH 5.5 was tested, expecting a significant growth arrest in the presence of GraXRS-blocking compounds. Following a similar approach, the dose–response behavior of selected compounds was evaluated. To do so, both bacterial growth and *dltxP* transcriptional activity were quantitatively assessed in the presence of variable concentrations, ranging from 0 to 20 μM, of selected drugs (Fig. 2C).

In order to reinforce the involvement of GraXRS TCS in the phenotypic outcomes rendered by the drugs to be chosen, their ability to down regulate the GraXRS-dependent alternative *mpr*F promoter was considered as an additional selective criterion.

To verify the specificity of GraXRS for the selected compounds, we restored a different TCS, *saeRS*, into the ∆XV genome. In this case, the reporter *lac*Z gene was transcriptionally fused to *sec*4 promoter, which has previously described as part of SaeRS regulon^22^. No shift in the transcription levels of *sec4*P::*lac*Z fusion appeared when ∆XV Sae-RES were incubated in the presence of each selected compound.

Finally, and though equivalency between wild type and GraXRS restored strains in terms of GraXRS-mediated sensing had been previously validated, the possibility that other TCSs or derived circuits absent on ∆XV Gra-RES could somehow affect *dltX* transcriptional activity was considered. In order to address this issue, the MW2 wild type strain was transformed with the *dlt*X-derived reporter plasmid and beta-galactosidase activity was measured in the absence and presence of mentioned compounds.

As shown in Fig. 2, the whole consecutive screening process significantly restricted the number of active compounds from almost 80 to 5. Acetylsalicylic acid (ASA), hesperidin (HES), ascorbic acid (VITC), verteporfin (VER) and troglitazone (TGZ) were capable of inhibiting bacterial growth under acidic conditions, affected *mprFP* activity negatively and exerted a suppressing effect on *dltXP* in the wild type genetic background. Among this final group of drugs, TGZ displayed an additional negative impact on SaeRS, and thus was firstly considered as a potential multi-target drug. However, since this molecule was withdrawn in 2000 due to high risk of hepatotoxicity, only ASA, HES, VITC and VER were contemplated as real candidates for therapeutic reposition. Curiously, though these four compounds show no common (known) pharmacological features, they are all chemically classified as redox-active drugs.

### In vitro effect of selected compounds: phagocytosis and killing of *S. aureus* by human PMNs

**Figure 3:**
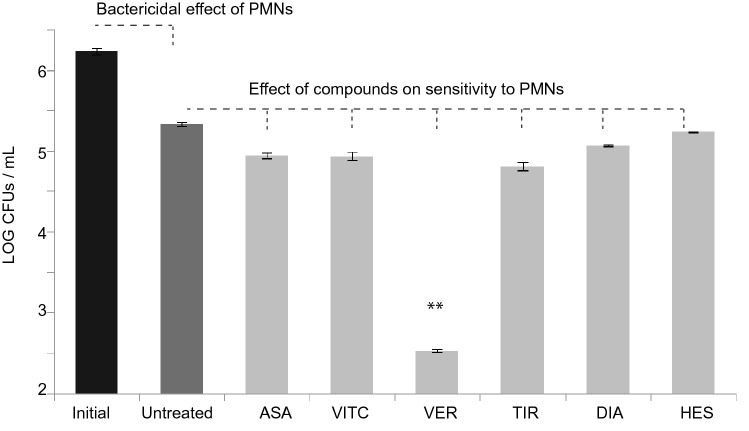
Effect of selected drugs in the susceptibility to phagocytosis and killing by human PMNs. Graphical scheme of bacterial counts expressed as log values after incubating PMNs and *S. aureus* MW2 wild type suspensions for 30 min in the presence or absence of four selected compounds (5 μM each), subsequent gentamicin treatments and lysis of eukaryotic cells (see “Materials and methods” section). Means and standard deviations are presented, illustrating the bactericidal effect of PMNs and the additional contribution of ASA, VITC, VER, TIR, DIA and HES. In the case of VER, two asterisks denote an associated p value of 0.006 when ANOVA and Tukey’s pairwise post hoc tests were applied according to prior normality and homoscedasticity tests.

Deepening into the anti-virulence potential of selected drugs, we next proceeded with an in vitro assay in which the bacterial susceptibility to phagocytosis and killing by human polymorphonuclear cells was assessed. Considering that GraXRS has been shown to have a pivotal role for *S. aureus* to resist PMN attack^20^, we isolated this cellular fraction from peripheral human blood and the effect of ASA, HES, VITC and VER on MW2 susceptibility to these immune cells was assessed. In all cases the process was boosted by opsonic antibodies naturally present in human sera due to unavoidable exposure to *S. aureus.* After incubating PMNs-*S. aureus* MW2 suspensions for 30 min in the absence or presence of the four selected compounds (5 μM each), removal of extracellular bacteria via gentamicin exposure, and subsequent lysis of eukaryotic cells at basic pH, bacterial viability was estimated via plate counting. Data shown in Fig. 3 prompted us to conclude that the presence of ASA, HES and VITC caused a slight increase in the sensitivity to PMN attack, while the effect of VER on reducing the number of surviving intracellular bacteria was substantially obvious and statistically significant (Fig. 3). As a result, from this in vitro approach, we chose VER as the sole candidate to proceed with the next assays.

### Effect of verteporfin in a murine model of wound infection

Following on from the in vitro testing, we next evaluated VER using the murine model of wound (or surgical site) infection. In this in vivo approach, a silk suture contaminated with *S. aureus* MW2 or MW2Δ*gra*X*RS* strain (4.5 × 10^5^ cfus cm^−1^) was used for sewing up a previous incision on the back of the mice. VER was topically applied 2 and 24 h after suture implantation using hydrogel-based formulations that contained either no active ingredient or the porphyrin under study at a low (0.125 mg/kg) or a high (2.5 mg/kg) dose. Assessment of the infection was performed by counting viable bacteria in tissue homogenates that were obtained upon animal euthanasia, 24 h after the last treatment.

**Figure 4:**
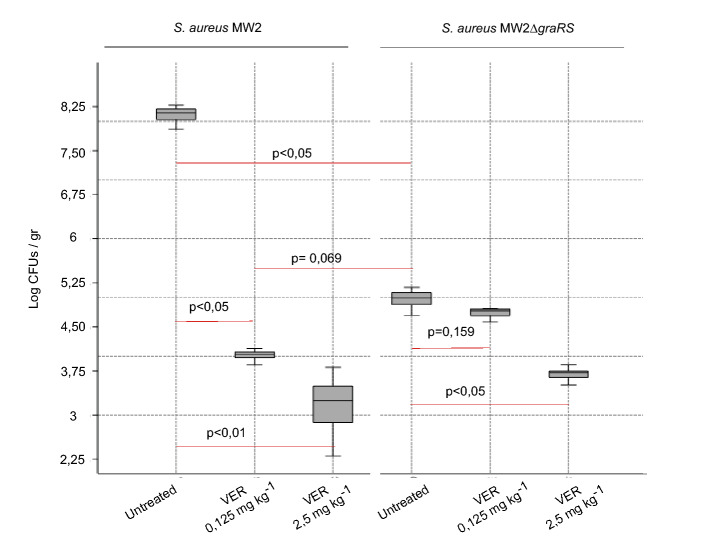
Effect of VER in a murine model of surgical wound infection. BOX-PLOT illustration (PAST statistical package) of bacterial counts expressed as log values per gram of wound tissue homogenates. Different treatments include the topical administration of hydrogel-based formulations with two doses (0.125 and 2.5 mg kg^−1^) of VER, 2 and 24 h after making an incision that was immediately infected with a surgical suture contaminated with 4 × 10^5^ CFUs of *S. aureus* MW2 wild type or ∆*graXRS* strain. Hydrogel without any active pharmaceutical ingredient was applied to control mice. All the animals were euthanized 24 h after the last treatment. Apart from visual interpretation in accordance to IQR overlapping, data were statistically analyzed via Kruskal–Wallis and post hoc Mann–Whitney test. Corrected p-values corresponding to relevant post hoc statistical comparisons are also shown.

As shown in Fig. 4, the significantly lower quantity of viable bacteria present in tissue samples that had been infected with the GraXRS deficient strain unveiled the critical role of this TCS in surgical wound infections. When the effect of VER was assessed, data showed that topical administration of this drug significantly reduced the bacterial load in a dose-dependent manner. Noticeably, application of VER at a low dose led to a similar degree of bacterial colonization to that followed by the implantation of sutures that had been contaminated with the GraXRS lacking strain. While such a result reinforces the potential of VER as a GraXRS inhibitory drug, its specificity is suggested by the fact that the observed outcome after treatment of wounds infected with the wild type strain is certainly evident but it becomes almost imperceptible in the case of ∆*gra*XRS-associated infection. Equally certain, however, is that high doses of VER could exert a GraXRS-independent and/or antibiotic effect.

### Exploring the mechanistic basis of verteporfin

**Figure 5:**
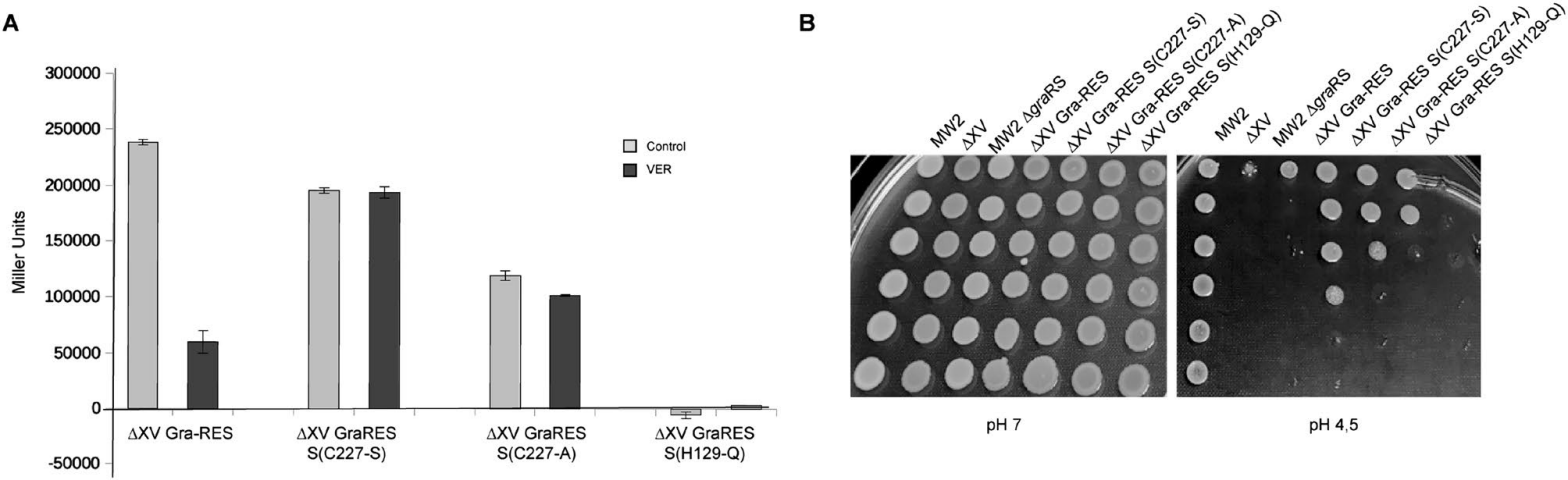
C227-mediated redox switch involvement in the sensitizing effect exerted by VER. (**A**) Transcriptional activity of GraXRS-dependent *dlt*X promoter in *S. aureus* ∆XV Gra-RES*,* ∆XV Gra-RES S(C227-S), ∆XV Gra-RES S(C227-A) and ∆XV Gra-RES S(H129-Q) strains is illustrated both in the presence (dark grey bars) or absence (light gray bars) of VER (10 μM). Means and standard deviations values are shown form at least three independent experiments. (**B**) Pictures illustrating the capacity of serially-diluted suspensions of bacterial strains *S. aureus* MW2 wild type, ∆XV, ∆*gra*RS, ∆XV Gra-RES, ∆XV Gra-RES S(C227-S), ∆XV Gra-RES S(C227-A) and ∆XV Gra-RES S(H129-Q) to grow in neutral (left) and acidified (right) TSA medium are also included in this figure.

Because VER is a tetradentate chelating porphyrin^23^ that might be involved in redox sensing, just like heme complex, and such molecules are normally sensed through thiol-based switches, we also analyzed the contribution of the single redox-active cysteine present in GraS, C227, to VER effect^24^. To do so, C227 was mutated to S or A in ∆XV Gra-RES background and transcriptional activity of *dltXP* in the resulting ∆XV Gra-RES S(C227-S) and ∆XV Gra-RES S(C227-A) strains was measured. An additional strain in which GraS H129 amino acid, the residue that undergoes phosphorylation upon activation of the kinase, had been mutated to Q was also constructed and included as a reference of complete GraXRS inactivation. As shown in Fig. 5A, data verified that replacement of cysteine by another residue had a negative impact on GraXRS activity, being such an outcome dependent on the polarity of the substituted amino acid. Thus, C227-A (non-polar) GraS isoform led to a lower degree of transcriptional activity of *dltXP* in comparison to that showed by the isoform in which C227 had been mutated to the polar amino acid serine. In accordance with this observation, bacterial growth arrest under acidic conditions, a phenotype that strictly reflects GraXRS status, also showed dependence on the mutation polarity (Fig. 5B). When the effect of VER was assessed, transcriptional data revealed that GraXRS repression led by mutations was far from being as drastic as the one achieved by VER (Fig. 5A), discarding the possibility of considering the C227 redox switch as the exclusive mechanism underlying VER effect. However, impairment of the signaling via C227 resulted in the insensitivity to VER, fact that suggests that intermolecular cysteine-disulfide-bond formation is required, though not entirely, for VER to have a blocking GraXRS-dependent effect.

## Discussion

Current strategies aimed at antimicrobial discovery prioritize innovative concepts like alternative molecular mechanisms of action, new natural product sources, pro-drugs, or even approved compounds that were originally intended for other therapeutic indications, as it is the case of the present work (Statement of Antimicrobial drug discovery, EASAC-2014^25^). Particularly speaking of *S. aureus,* and discarding the essential TCS WalKR/S as the antibiotic target *per excelence*, continuous efforts are being made toward the discovery of inhibitors of TCS involved in virulence and biofilm formation (for review^26–28^). Impairment of these non-essential biological pathways has the advantage of requiring a lower plasmatic dose compared with a MIC, reducing the tendency to resistance and minimizing side effects on neutral and beneficial microbiota that colonize treated human or animal hosts^29^. Up to date, several novel and previously approved drugs with the capacity to exert an inhibitory effect on Agr, SaeRS, and ArlRS TCSs have been described (for review^26–28^) but, to our knowledge, GraXRS had never been considered as a target for disarming *S. aureus*. The involvement of this TCS in the resistance to antimicrobial peptides and macrophages had already been envisioned in several occasions^9,30,31^, but it has just been recently proven that GraXRS is entirely responsible for the response to pH inside acidified macrophage phagolysosomes^15,20^. These premises led us to consider GraXRS as a clear target to counteract *S. aureus* response to innate host immunity and impede replication of the pathogen in the acute stage of systemic infection.

Taking advantage of the *S. aureus* strain that lacks its complete sensorial TCS network^15^, we developed a series of reporter strains that could be helpful for selecting compounds capable of blocking transcriptional activity of the GraXRS-dependent *dltX* promoter. Working with ∆XV Gra-RES strain gave us the opportunity to perform a bioassay where bacterial sensing entirely depends on GraXRS (and WalkRS), thus reducing the probability of selecting off-target drug candidates. Proof of this last claim is the fact that among selected drugs, only one of them (Troglitazone) displayed a GraXRS-SaeRS multi target effect.

At the time of deciding the type of compound to be tested for the identification of GraXRS inhibitors, we, as many other researchers, opted for the drug-repurposing approach. This strategy is based on the identification of “off” antimicrobial targets for drugs that were approved for other clinical diseases^29^, hence bypassing the financial and regulatory barriers that have to be overcome to bring a drug to market. At present day, this concept of repurposing has gained renewed interest and a novel category of drugs known as “Potential Drugs for Repurposing against Infectious Agents” is exponentially thriving^32^ By way of example, the old antimalarial drug chloroquine is being tested as SARS-CoV-2 inhibitor^33^. Though the precise mechanistic basis of their effect remains to be completely elucidated, current candidate PDRIAs targeting *S. aureus* SaeRS and/or AgrTCSs are floxuridine, streptozotocin and diflunisal^28,34,35^.

The screening methodology applied here consisted in the analysis of changes in *dltXP* transcriptional activity, followed by several consecutive steps where additional criteria like the effect on an alternative GraXRS-dependent promoter, TCS-selectivity, or the determination of bacterial growth in the presence of selected drugs under acidic conditions were applied. The overall process ended up in the selection of five candidate drugs: acetylsalicylic acid (ASA), hesperidin (HES), ascorbic acid (VITC), verteporfin (VER) and troglitazone (TGZ). Curiously, all compounds are classified as redox-active drugs. While ASA, HES, VITC and TGZ are commonly sorted as antioxidant molecules, VER can induce oxidative stress through the production of free radicals or be alternatively combined with soluble metals and display a redox potential similar to that showed by the heme complex^23^. These observations are in agreement with the previously unveiled connection between GraXRS and oxidative stress, evidenced by the deciphering of the GraXRS regulon and the proved essentiality of this TCS in staphylococcal resistance to redox compounds like paraquat or H_2_0_2_^9^. Furthermore, a recent RNA-seq transcriptomic approach has just corroborated the involvement of GraXRS, collectively with VraSR, SaeRS, MgrA, SigB or Fur, in the cell response to thiol-oxidative stress^36,37^.

After proving that VER was the only compound really capable of sensitizing bacteria against the effect of human PMNs, this compound was further examined using a murine model of surgical wound infection. This model has been previously used to assess the effect of systemic and topical antimicrobial agents, finding a close correlation with efficacy in clinical trials with human subjects. Noticeably, our results provided further evidence concerning the critical role of GraXRS in skin and wound infections and insinuated a pharmaceutical potential of VER as a novel local treatment for S. *aureus* infections. Since bacterial count after the infection with the wild type strain and subsequent treatment with 0.125 mg kg^−1^ of VER was quite similar to that proceeding from the infection with a GraXRS negative strain, the effect exerted by the porphyrin derivative seems to be highly dependent on the activity of this TCS.

When the possible path(s) of how VER inhibits the activity of GraXRS was envisaged, we thought of VER as a heme-like porphyrin capable of binding iron in different oxidation states^23^. Recent transcriptomic studies conducted with the constitutively-active forms of staphylococcal kinases have unveiled the involvement of GraXRS in the regulation of heme synthase A (MW_RS05355;^38^), fact that led us to attach importance to the chemical mimicry between both molecules. Since porphyrins are normally involved in thiol-based molecular switches^24^, the unique potentially redox-active residue in GraS, C227, was considered a potential molecular target of VER. To address this question, the impact of C227 mutations to S and A on GraXRS activity and VER sensitivity was assessed. To our knowledge, the results achieved in this work by punctual mutations of C227 have evidenced for the first time that this cytosol-located redox-active residue actually participates in GraS kinase activity. This line of reasoning, which has support from studies that have characterized other bacterial kinases like AcrB^39^, showed that GraS degree of silencing depended on the polar nature of the amino acid that substituted C227 and suggested the involvement of this redox-switch as a potential molecular target concerning VER effect. However, and though C227 substitutions led to insensitivity to VER, the inhibitory outcome yielded by mutations was not as drastic as the one achieved by exposure to VER, suggesting that additional molecular paths must be involved in this process. An additional candidate that might be considered is Stk1, the unique serine-threonine kinase which cross-phosphorylates GraR^40^ and shows homology with OXR1, one of the recently discovered mammalian target of VER that, curiously, is also related to oxidative stress^41^. However, in accordance with Fridman et al.^40^, we have verified that both Stk1 and GraS-mediated phosphorylation on GraR as T128, T130, T149 and D51 respectively are equally required for full *dltX* expression (data not shown), fact that seriously complicates the use of our reporter systems when it comes to holding Stk1 accountable for intervening on VER effect.

Could VER be considered a viable antimicrobial candidate? Though we are fully aware that further in vitro and in silico studies helping to understand the whole molecular scenario underlaying VER effect and alternative in vivo approaches or definition of strategic dosages are some pending issues to claim a novel anti-virulence pharmaceutical indication for VER, we are convinced that this drug presents some strengths, apart from those inherent to anti-virulence drugs, that might be worth considering. In terms of pharmacology, for instance, VER excretion is dependent on hepatic function, while many antibiotics primarily undergo elimination via kidney filtration. Thus, VER could be administered to patients suffering from kidney disease, or to those who were being concomitantly treated with antibiotics that are prone to cause nephrotoxicity (e.g. vancomycin)^42^. On the other hand, though side effects associated to parenteral administration of VER include hypersensitivity reactions or blood pressure alteration, these symptoms have lower mean severity ratings in comparison to those showed by many antibiotics (ema.europa.eu/Find medicine/Human medicines/European public assessment reports). Finally, taking into account that we have also observed that VER-containing topical formulations are effective and that further studies might confirm that chemical mimicry between porphyrin derivatives and heme group could actually be harnessed for disarming *S. aureus,* our results may be considered as a step forward in re-proposing VER as a plausible alternative in combating antimicrobial resistance.

## Materials and methods

### Bacterial strains, plasmids, oligonucleotides and culture media

Bacterial strains, plasmids and oligonucleotides (purchased to IDT) are listed in Tables 1, 2 and 3 respectively. *Escherichia coli* XL1blue strain was grown in LB broth and LB agar. *Staphylococcus aureus* strains were grown on trypticase soy broth (TSB), trypticase soy agar (TSA), trypticase soy broth with 0.2% of glucose (TSB-glu), Mueller Hinton (MH) and B2 medium [1% casein hydrolysate, 2.5% yeast extract, 2.5% NaCl, 0.1% K_2_HPO_4_, and 0.5% glucose (w/v)]. When required for growth or selection, medium was supplemented with 5-Bromo-4-Chloro-3-Indolyl β-d-Galactopyranoside (XGal) and/or the appropriate antibiotic at the following concentrations: erythromycin (Eri) 10 μg ml^−1^, ampicillin (Am) 100 μg ml^−1^, chloramphenicol (Clo) 10 μg ml^−1^ and 20 μg ml^−1^.

**Table 1:** Strains used in this study.

Strain	Characteristics	References
***Escherichia coli***		
Xl1Blue	Cloning strain (*recA1 endA1 gyrA96 thi-1 hsdR17 supE44 relA1 lac* [F′ *proAB lacI*q*Z*∆*M15* Tn*10* (Tetr)])	Stratagene
***Staphylococcus aureus***		
RN4220	Restriction deficient transformation recipient	^48^
MW2	Community-acquired strain of MRSA isolated in 1998 in North Dakota, USA	^49^
MW2Δ*gra*RS	Markerless mutation of *graRS* genes	^15^
ΔXV	MW2 *∆hptRS ∆lytSR ∆graRS ∆saeRS ∆MW1208-MW1209 ∆arlRS ∆srrAB ∆phoPR ∆yhcSR ∆vraSR ∆agrBDCA ∆kdpDE ∆hssRS ∆nreBC ∆braRS*	^15^
ΔXV Gra-RES	Restored ΔXV::*gra*RS strain	This study
ΔXV Gra-RES S(C227-A)	Restored ΔXV::*gra*RS strain with C227-A single amino acid substitution in GraS	This study
ΔXV Gra-RES S(C227-S)	Restored ΔXV::*gra*RS strain with C227-S single amino acid substitution in GraS	This study
ΔXV Gra-RES S(H129-Q)	Restored ΔXV::*gra*RS with H129-Q single amino acid substitution in GraS	This study
ΔXV Gra-RES Δ*gra*X	Markerless mutation of *graX *in ΔXV Gra-RES strain	This study
ΔXV Sae-RES	Restored ΔXV::saeRS strain	This study
MW2 *mpr*Fp	MW2 carrying pSA14::*mprF*p; Eri^R^	This study
MW2 *dlt*Xp	MW2 carrying pSA14::*dlt*Xp; Eri^R^	This study
MW2 *sec4*p	MW2 carrying pSA14::*sec4*p; Eri^R^	This study
ΔXV *mpr*Fp	ΔXV carrying pSA14::*mpr*Fp; Eri^R^	This study
ΔXV *dlt*Xp	ΔXV carrying pSA14::*dltX*p; Eri^R^	This study
ΔXV *sec4*p	ΔXV carrying pSA14::*sec4*p; Eri^R^	This study
ΔXV Gra-RES *mpr*Fp	Restored ΔXV::*gra*RS strain carrying pSA14::*mpr*Fp; Eri^R^	This study
ΔXV Gra-RES *dlt*Xp	Restored ΔXV::*gra*RS strain carrying pSA14::*dlt*Xp; Eri^R^	This study
MW2 Δ*gra*RS *mpr*Fp	MW2 Δ*gra*RS carrying pSA14::*mpr*Fp Eri^R^	This study
MW2 Δ*gra*RS *dlt*Xp	MW2 Δ*gra*RS carrying pSA14::*dlt*Xp Eri^R^	This study
ΔXV Sae-RES *sec4*p	Restored ΔXV::*Sae*RS carrying pSA14::*sec4*p Eri^R^	This study

**Table 2:** Plasmid used in this study.

Plasmid	Characteristics	References
pMAD	*E. coli–S. aureus* shuttle vector with a thermosensitive origin of replication used for allelic replacement	^45^
pMAD::*gra*RES	pMAD plasmid containing the allele for chromosomal restoration of *gra*RS TCS	This study
pMAD::*sae*RES	pMAD plasmid containing the allele for chromosomal restoration of s*ae*RS TCS	This study
pMAD:: ∆*gra*X	pMAD plasmid containing the allele for markerless deletion of *gra*X gene	This study
pMAD::*gra*RES S(C227-A)	pMAD plasmid containing the allele for chromosomal restoration of *graRS* S(C227-A) isoform	This study
pMAD::*gra*RES S(C227-S)	pMAD plasmid containing the allele for chromosomal restoration of *graRS* S(C227-S) isoform	This study
pMAD::*gra*RES S(H129-Q)	pMAD plasmid containing the allele for chromosomal restoration of *graRS* S(H120-Q) isoform	This study
pSA14	pM4 derivative carrying the promoterless *E.coli lac*Z gene for constructing transcriptional fusions	^9^
pSA14::*mpr*Fp	pSA14 containing the *mpr*F promoter region	^9^
pSA14::*dlt*Xp	pSA14 containing the *dlt*X promoter region	This study
pSA14::*sec*4p	pSA14 containing the *sec4* promoter region	This study

**Table 3:** Oligonucleotides used in this study.

Oligonucleotide	Sequence
**Restoration of TCS**	
gra-E	GGGCCATAAAAAGCCTCCAG
gra-F	GTAGCTTCCGACTTGTGAGCC
gra-A (EcoRI)	CCGGGAGCTCGAATTCCAAATAGATATTGCTGTATTCTTTATCGACCCAAC
gra-D (BamHI)	GGGCGATATCGGATCCAACGCCACCTAAAACACTTTGTACAC
G C227A Rv	CTAATAATCATACGGCACCATTTTATATC
H C227A Fwd	GATATAAAATGGTGCCGTATGATTATTAG
G C227S Rv	TCTAATAATCATACGAGACCATTTTATATC
H C227S Fwd	AGATATAAAATGGTCTCGTATGATTATTAG
G H129-Q Rv	GTTTTTATGTCTTGCACAAATTCTG
H H129-Q Fwd	CAGAATTTGTGCAAGACATAAAAAC
sae-E	AGTACAATTTGATGATGGTGTTGGTG
sae-F	GATTTCACAGCACCCCTAGC
sae-A (BamHI)	GGGCGATATCGGATCCCAAAAGGGTTATTTGAATGGATAGGC
sae-D (NotI)	CCATGGCATGCATCGCTGTTCACATAACACTACAAATCGC
graX A (BglII)	CACAGATCTGGTTGGTTATTGAGTGGTACATTTG
graX B (XhoI)	CACCTCGAGCTAAAATACTCCTTTAAACTGTAACC
graX C (XhoI)	CACCTCGAGGGTGATATGGATGCAAATAC
graX D	GGAGGATCCTTTCGATTTGATTTTTTTTGGTAATAAG
graX E	GTTGTTATGCGATTCTGATACAAG
graX F	TGTTTCGATTGCACTATCCATAC
**Reporter construction**	
pSA14-Fw	TGGAATTGTGAGCGGATAAC
pSA14-Rv	CTCTTCGCTATTACGCCAG
mprFp-Fw (PstI)	CTGCTGCAGTATAGATAACCATATTGTTC
mprFp-Rv (BamHI)	GGAGGATCCTGATTCATTTTTTCACATCA
dltXp-Fw (PstI)	GGCTGCAGGCGCTGATGATAATTCAATAA
dltXp-Rv (BamHI)	CGGGATCCGATTTCATATTGCACCTCTTAAAG
sec4p-Fw (PstI)	GGCTGCAGGAGTGTGAATATATAAACAATG
sec4p-Rv (BamHI)	GCGGATCCTTATTCATTTTTATCTCCTTC
mprFp-GFP-Fw (SalI)	GTCGTCGACGTATAGATAACCATATTGTTC
mprFp-GFP-Rv (KpnI)	GGTGGTACCTGATTCATTTTTTCACATCA
**Complementation plasmid**	
graR Fw (KpnI)	GGGGTACCTCGAGAATGATATTGGGTGATATGG
graR Rv (EcoRI)	GGGAATTCCAAATTATTCATGAGCCATATA

### DNA manipulations

Plasmids were purified using NucleoSpin Plasmid kit (Macherey–Nagel) according to the protocol provided by the manufacturer. PCR fragments and enzymatic reactions were purified using GeneJET Gel Extraction and DNA Cleanup Micro Kit (Thermo Scientific). FastDigest restriction enzymes, Rapid DNA ligation kit, Dreamtaq DNA polymerase and Phusion DNA polymerase were supplied by Thermo Scientific and used according to provided instructions. Sequence verification of PCR-amplified products and plasmid constructions was performed by Stab Vida. Transformation of *Staphylococcus aureus* was performed following previously standardized protocols^43,44^.

### Allelic exchange of chromosomal genes

To generate markerless deletions, two fragments of at least 500 bp that flanked upstream (primers A and B, Table 3) and downstream fragments (primers C and D, Table 3) of the region to be deleted were amplified by PCR. Amplified products were digested using the corresponding restriction enzymes (Table 3), purified and cloned by ligation into pMAD shuttle vector. To restore individual TCSs into ∆XV chromosome, a fragment containing the two flanking regions used to generate the deletion^15^ and the original TCS sequence were amplified by PCR using chromosomal DNA from MW2 strain as template and oligonucleotide pair *gra* or *sae* 1–3. For restoration of the TCS with single amino acid substitutions at C227, *gra*RS was amplified using MW2 chromosomal DNA as template and a two-step PCR protocol. First, the oligonucleotides A and G or H were used for generating two overlapping PCR products, while a second amplification step with A and D oligonucleotides using both purified PCR products as templates generated *gra*RS S(C227-A) and *gra*RS S(C227-S) isoforms. Such DNA fragments were purified, digested with the corresponding enzymes (see Table 3) and inserted by ligation into the pMAD shuttle vector^45^. Homologous recombination experiments were performed as previously described^46^. Final plasmidless erythromycin sensitive white colonies were tested by PCR using primers E and F (Table 3).

### Reporter plasmid construction

pSA14 was used for the construction of different reporter plasmids. Promoter regions of *mprF*, *dltX* and *sec4* were amplified by PCR using oligonucleotides described in Table 3. PCR fragments were purified and cloned into pSA14 through restriction enzymes to generate transcriptional fusion with *lacZ*. GraRS-dependent reporter plasmids were transformed via electroporation into *S. aureus* MW2, MW2Δ*gra*XRS, ΔX, ΔXV Gra-RES and ΔXV Gra-RESΔ*gra*X, while plasmid harboring Sae-dependent *sec4* promoter was inserted into MW2, ΔXV and ΔXV Sae-RES strains. To analyze *mprF, dltX* and *sec4* expression, ON cultures were chemically lysed beta-galactosidase activity was measured.

### High throughput beta-galactosidase-based screening

Screening of the 1280 off-patent FDA-approved drugs in Prestwick Chemical drug library (Prestwick Chemical) was based in a method for beta-galactosidase assays in 96 well plates. Working solutions of the compounds were prepared in 50 μl of sterile distilled-deionized water at a concentration of 20 μM, and combined with 50 μl of a 1:30 dilution on 2 × TSB medium of an overnight (ON) culture of *S. aureus* strains, thus generating 100 μl of 1:60 cell dilution on 1 × TSB at a final concentration of 10 μM of each drug. Plates were incubated during 24 h at 37 °C and, upon incubation, OD_600nm_ was measured (Multiskan Go; Thermo Scientific). Bacterial cells were subsequently lysed by the addition of 100 μl well^−1^ of Z buffer supplemented with lysostaphin (0.5 mg ml^−1^) during 2 h at 37 °C; Next, 30 μl well^−1^ of Ortho-Nitrophenyl-beta-galactoside (ONPG, 4 mg ml^-1^) was added and, when required, the reaction was stopped with 100 μl well^-1^ of 1 M Na_2_CO3. OD_420_ and OD_550_ values were finally recorded for Miller Units calculation. Untreated reporter ∆XV-GraRES and ∆XV strains were included in every plate as internal controls. Experiments were carried out in triplicate.

### Phagocytosis and killing of *S. aureus* by human PMNs

Phagocytosis and killing of *S. aureus* by human neutrophils in presence of selected compounds was determined as described before^47^. Polymorphonuclear cells (PMNs) were isolated from healthy human heparinized-defibrinated blood (Seralab Logistics) using Ficoll-Plaque PREMIUM (GE-Healthcare) according to manufacturer’s protocol and resuspended at a final concentration of 1 × 10^7^ PMNs ml^−1^ in HBSS supplemented with human serum. *S. aureus* strains were cultured to the early stationary phase and 10 ml of culture were centrifuged, washed twice with sterile PBS and resuspended in Hank’s Balanced Salt Solution (HBSS) supplemented with human serum at a final concentration of 4 × 10^5^ bacteria ml^−1^. Finally, 0.2 ml of PMNs solution was mixed with 0.2 ml of *S. aureus* solution and 600 μl of HBSS supplemented with human serum. Compounds were added at a final concentration of 5 μM. After incubation at 37 °C for 30 min, each sample was treated with gentamicin 100 mg ml^−1^ and then 100 μl of each mixture were added to 1 ml of pH 11 solution. Finally, serial dilutions were plated on TSA to determine the number of colony-forming units (CFU) in presence of the different compounds. All data were referred to initial CFU number.

### Mouse infection model

The experimental animal study was reviewed and approved by the “Comité de Ética, Experimentación Animal y Bioseguridad” of the Universidad de Navarra-Centro de Investigación Médica Aplicada (CIMA). Work was carried out at the CIMA animal facility under the principles and guidelines described in the “European Directive 86/609/EEC” for the protection of animals used for experimental purposes. Six-week-old female Swiss mice (20–25 g) were obtained from ENVIGO and confined in groups of 6 animals.

The model was performed as previously described^50^. Briefly, 10 cm fragments of commercial brailed silk (TC-15, Lorca Marín) were contaminated with 4 × 10^6^ CFU cm^−1^ of *S. aureus* MW2 or *S. aureus* MW2Δ*gra*XRS strains by immersion during 30 min. Fragments were then blotted- dried. One day prior to the experiment, the interscapular skin was shaved using a sharp razor. On the day of the infection, superficial wounds were produced on the exposed back surface though a longitudinal midline incision of 2 cm approximately. The skin of either side of the incision was retracted, and the wound was infected by stitching it with contaminated suture and a suturing needle. Wounds were topically treated 1 h and 8 h after infection with approximately 100 μl of hydrogel formulations containing 0.125 and 2.5 mg/100 μl. The hydrogel base without any active substance was applied in the control group. Treatments were repeated 24 h after infection and mice were sacrificed 24 h after the last application. Finally, the wounded tissue was resected, homogenized in PBS, and dilution series of homogenates was plated on TSB agar for enumeration of CFU (output). After an overnight incubation at 37 °C, CFU gr of tissue^-1^ were calculated and expressed as log_10_.

### Statistical analysis

Data generated by PMN-mediated killing assay were compared using ANOVA, applying Tukey’s pairwise as post hoc test. Data obtained from the bacterial counts in the murine model were treated and compared using Kruskal–Wallis test, Mann–Whitney pairwise and Dunn’s post hoc tests. All tests were two-sided, and the significance level was 5%. The statistical analysis was performed with Past and R softwares.

## Supplementary information

Supplementary Table 1.
