# Supplementary material for: Inhibiting the two-component system GraXRS with verteporfin to combat Staphylococcus aureus infections
Source: Sci Rep. 2020 Oct 21;10:17939. doi: 10.1038/s41598-020-74873-5 (PMC7577973; doi:10.1038/s41598-020-74873-5)
Supplement: Supplementary file 1 — Supplementary Table 1. [file 41598_2020_74873_MOESM1_ESM.pdf]

Inhibiting the Two-Component System GraXRS with Verteporfin to combat *Staphylococcus aureus* infections

Juana María Prieto[1], Beatriz Rapún-Araiz[2], Carmen Gil[2], José R. Penadés[3], Iñigo Lasa[2] and Cristina Latasa[1*]

Supplementary information

Supplementary table 1: Compounds screened and their effect on *gra*XRS transcriptional activity

Name	Therapeutic effect	% GraRS inhibition	% Growth inhibition
Neomycin sulfate	Antibacterial	180,15	39,79
Promazine hydrochloride	Antipsychotic	-455,16	56,7
Econazole nitrate	Antifungal	173,73	50,6
Ascorbic acid	Antioxidant CNS Stimulant Hemostatic	132	18,77
Chlorphensin carbamate	Muscle relaxant	117,88	8,82
Hesperidin	Anti-haemorrhoids Antineoplastic Antioxidant	115,43	9,86
Nicergoline	Anti-ischemic vasodilator	108,5	49,62
Nalbuphine hydrochloride	analgesic	99,03	50,25
Acetylsalicylic acid	Analgesic Anti-inflammatory Antipyretic	89,91	8
Ornidazole	Antibacterial antiparasitic antiprotozoal	89,65	25,16
Tazobactam	Antibacterial	-383,54	60,19
Clomiphene citrate (Z,E)		85,25	53,63
Streptomycin sulfate	Antibacterial	83,29	29,18
Troglitazone	Antidiabetic anti-inflammatory	79,86	48,71
Daunorubicin hydrochloride	Antibacterial antineoplastic	70,01	30,82
Thioridazine hydrochloride	Antipsychotic	67,57	-26,73
Oxantel pamoate	Antihelmintic	66,59	6,44
Cefazolin sodium salt	Antibacterial	-1702,12	95,61
Triclabendazole	Antihelmintic	64,07	2,36
Scopolamin-N-oxide hydrobromide	Antispastic mydriatic	63,42	-1,86
Tiratricol, 3,3',5-triiodothyroacetic acid	Antihypothyroid hypocholesterolemic	60,48	34,92
Amidopyrine	Analgesic anti-inflammatory antipyretic	59,64	32,77
Aminocaproic acid	Antifibrionolytic Hemostatic	58,89	8,78
Trimethobenzamide hydrochloride	Antiemetic	55,32	-15,5
Azaguanine-8	Antineoplastic	53,86	4,14
Orphenadrine hydrochloride	Antihistaminic antiparkinsonian	47,19	-7,04
Sulindac	nalgesic anti-inflammatory antypyretic	45,8	-14,37
Busulfan	Antineoplastic	43,69	29,62
Flavoxate hydrochloride	Antispastic	43,25	2,87
Monensin sodium salt	Antibacterial	70,62	80,76
Hyoscyamine (L)	Antiemetic antispastic mydriatic	42,71	7,27
Diacerein	Antiarthritic	42,06	15,85
Amisulpride	Antipsychotic	-310,8	55,94
Cefpodoxime proxetil	Antibacterial	-306,94	72,71
Demeclocycline hydrochloride	Antibacterial	-69,98	89,49
Amitryptiline hydrochloride	Antidepressant	41,03	-40,03
Piretanide	Antihypertensive Diuretic	39,78	-2,32
Amprolium hydrochloride	anticoccidial antiparasitic	38,28	-13,2
Ethosuximide	Anticonvulsant	37,08	26,96
Paclitaxel	Antineoplastic	36,91	38,02
Diazoxide	Antidiuretic antihypertensive vasodilator	36,44	37,36

Name	Therapeutic effect	% GraRS inhibition	% Growth inhibition
Valproic acid	Anticonvulsant	36,39	5,2
Phenelzine sulfate	Antidepressant	36,16	33,49
Verteporfin		33,95	30,24
Imipramine hydrochloride	Antidepressant	32,51	-27,35
Clebopride maleate	Antiemetic Antispastic	31,2	30,25
Azacytidine-5	Antineoplastic	30,95	49,41
Adiphenine hydrochloride	Antispastic	30,73	-16,78
Sildenafil	Antihypertensive Erectile dysfunction treatment	30,69	20
Carprofen	Anti-inflammatory	30,38	24,96
Chlorpheniramine maleate	Antihistaminic antitussive sedative	29,77	50,15
Hydralazine hydrochloride	Antihypertensive	28,62	22,14
Prednisone	Anti-inflammatory antipruritic Immunosuppressant	27,07	-22,18
Acetazolamide	Anticonvulsant antiglaucoma diuretic	26,95	-8,17
Prednicarbate	Anti-inflammatory	26,56	-2,42
Clotrimazole	Antibacterial antifungal	26,55	15,35
Todralazine hydrochloride	Antihypertensive	25,89	-34,39
(-)-Emtricitabine	antiviral	-257,79	56,73
Alexidine dihydrochloride	Antibacterial	36,85	94,38
Riluzole hydrochloride	Antipastic Neuroprotectant	25,89	23,66
Chloropyramine hydrochloride	Antihistaminic	25,54	-4,78
Diphemanil methylsulfate	Antispastic antiulcer	25,17	-19,33
Isoflupredone acetate	Anti-inflammatory	23,83	-3,75
Ketoconazole	Antifungal	23,57	29,91
Diethylstilbestrol		23,25	35,06
Proglumide	Antiulcer	22,66	-0,42
Dicumarol	Anticoagulant	-523,22	74,71
Desonide	Antipsoriatic	20,05	-0,61
Merbromin	Antibacterial	-3277,68	92,27
Verapamil hydrochloride	Antihypertensive	19,94	35,51
Acyclovir	Antiviral	19,72	28,55
Cisatracurium besylate	Muscle relaxant	19,72	-6,16
Disulfiram	Antabuse effect	19,14	46,42
Cefprozil	Antibacterial	18,36	7,81
Tolfenamic acid	Analgesic anti-inflammatory	18,25	33,52
Etomidate	Anesthetic Hypnotic	18,05	3,23
Tioconazole	Antifungal	17,7	54,75
Nitrofurantoin	Antibacterial	17,5	26,14
Pemetrexed disodium	Antienoplastic	16,84	-0,68
Carbinoxamine maleate salt	Antihistaminic	16,48	-2,82
Silodosin	Antihypertenisve	16,33	0,44
Sulconazole nitrate	Antifungal	82,06	72,66

Name	Therapeutic effect	% GraRS inhibition	% Growth inhibition
Brompheniramine maleate	Antihistaminic antipruritic antitussive	15,57	26,91
Mitoxantrone dihydrochloride	Antineoplastic	15,07	23,47
Oxymetazoline hydrochloride	Nasal decongestant vasoconstrictor	14,94	39,45
Penciclovir	Antiviral	14,8	30,79
Dibucaine	Local anesthetic	14,78	-14,63
Spectinomycin dihydrochloride	Antibacterial	14,51	-1,78
Cytarabine	Antineoplastic	-241,17	58,32
Furazolidone		14	12,21
Moxalactam disodium salt	Antibacterial	-692,97	97,01
S-(+)-ibuprofen	Analgesic Anti-inflammatory	12,89	15,15
Auranofin	Analgesic	104,81	96,32
Indapamide	Antihypertensive diuretic	12,74	42,6
Hydrochlorothiazide	antihypertensive diuretic	12,24	-6,91
Metformin hydrochloride	anorectic antidiabetic antilipmic	11,91	5,61
Sulmazole	Cardiotonic	11,79	5,44
Oxybenzone		11,03	-5,77
Valsartan	Vasodilator Antihypetensive	10,84	-3,63
Repaglinide	Antidiabetic	10,38	4,34
Gallamine triethiodide	Muscle relaxant	10,12	21,75
Nefopam hydrochloride	Analgesic	9,68	40,78
Raltitrexed	Antineoplastic	9,63	-3,86
Suprofen	Analgesic Anti-inflammatory	9,36	13,93
Thyroxine (L)	Antihypothyroid antilipemic hypocholesterolemic	9,33	13,73
Tacrine hydrochloride	CNS Stimulant	8,78	22,66
Sulbactam	Antibacterial	8,61	4,65
Edrophonium chloride	Anti-fatigue	8,56	24,75
Brimonidine L-Tartrate	Antiglaucoma	8,43	2,22
Guanethidine sulfate	Antihypertensive Local anesthetic	8,05	4,75
Tridihexethyl chloride	Antispastic	6,59	-1,83
Mefenamic acid	Analgesic anti-inflammatory antipyretic	6,5	17,94
Ethynylestradiol 3-methyl ether		6,39	14,47
Penbutolol sulfate	Antianginal Antiarrhythmic Antihyperensive	6,29	4,36
Lithocholic acid	Cholangogue Choleretic	6,06	25,71
Novobiocin sodium salt	Antibacterial	113,09	83,58
Antimycin A	Antibacterial antifungal	5,46	45,65
Clobutinol hydrochloride	Antitusive	-223,43	58,87
Dichlorphenamide	Antiglaucoma	5,35	-1,62
Zoxazolamine	Antigout Muscle relaxant Uricosuric	5,22	6
Clinafloxacin	Antibacterial	62,07	77,42
Trimipramine maleate salt	Antidepressant	5,03	-2,92
Piromidic acid	Antibactierial	4,87	1,47

Name	Therapeutic effect	% GraRS inhibition	% Growth inhibition
Allantoin	Antipsoriatic Vilnerary	3,48	-5,66
Nafcillin sodium salt monohydrate	Antibacterial	-488,7	96,58
Hydroxyzine dihydrochloride	antiemetic antihitaminic antipruritic	3,28	46,1
Rofecoxib	Anti-inflammatory	2,98	8,5
Dipyridamole	Anticoagulant antiplatelet vasodilator	2,31	45,14
Phenylpropanolamine hydrochloride	Antihypotensive Nasal Decongestant Vasoconstrictor	2,3	17,48
Miconazole	Antifungal	2,1	53,1
Milnacipran hydrochloride	Antidepressant Analgesic	2,07	4,57
Dilazep dihydrochloride	Antiplatelet vasodilator	1,74	14,01
Amiloride hydrochloride dihydrate	antihypertensive diuretic	1,7	-4,42
Chlorzoxazone	Anticonvulsant Muscle relaxant	1,69	23,43
Canrenoic acid potassium salt	Antihypertensive diuretic	1,47	49,88
Roxithromycin	Antibacterial	107,08	81,98
Methazolamide	Antiglaucoma Diuretic	1,41	-2,55
Bisoprolol fumarate	Antianginal Antiarrhythmic Antihypertensive	1,11	10,04
Atracurium besylate	Curarizing	0,78	-4
Sulfamethoxypyridazine	Antibacterial	0,78	11,18
Haloprogin	Antifungal	0,46	47,63
Tyloxapol	Mucolytic	0,15	-3,13
Oxyphenbutazone	Anti-inflammatory	-0,11	1,48
Troxipide	Antiulcer	-0,67	3,9
Gestrinone	Contraceptive	-0,95	-4,9
Methyldopa (L,-)	Antihypertensive	-1,5	23,67
Ubenimex	Antineoplastic Immunomodulator	-1,83	8,18
Indinavir sulfate	Antiviral	-2,1	12,26
Pyrithyldione	Hypnotic Sedative	-2,36	-0,49
Loracarbef	Antibacterilal	-208	64,86
Etoposide	Antineoplatic	-2,42	31,84
Gemfibrozil	Hypocholesterolemic lipid-lowering	-2,79	31,14
Testosterone propionate	Anabolic	-3,23	9,71
Albendazole	Antihelmintic antiparasitic	-4,56	12,37
Doxapram hydrochloride	Analeptic Eupneic	-4,89	5,08
Idebenone	Antineoplastic	-5,13	7,44
Amorolfine hydrochloride	Antifungal	-6,16	8,28
Meglumine	Antileishmanial Antiseptic Expectorant	-6,55	-2,24
Metronidazole	Antiamebic antibacterial antiprotozoal	-6,87	43,47
Flufenamic acid	Analgesic anti-inflammatory antipytetic	-7,92	35,14
Trifluoperazine dihydrochloride	Antiemetic antipsychotic	-8,18	12,76
Carbamazepine	Analgesic anticonvulsant antidiuretic	-9,02	15,35
Denatonium benzoate		-9,11	-2,3
Esmolol hydrochloride	Antiarrhythmic	-9,52	3,08

Name	Therapeutic effect	% GraRS inhibition	% Growth inhibition
Carbachol	Antihypertenisve Vasodilator	-9,83	8,81
Pravastatin	Antilipemic	-10,01	17,7
Ketanserin tartrate hydrate	Antihypertensive Vasodilator	-10,37	0,68
Carbenoxolone disodium salt	Antiulcer	-10,37	31,75
Digoxigenin		-10,63	-1,84
Quinethazone	Antihypertensive Diuretic	-11	8,13
Rabeprazole Sodium salt	Antiulcer	-11,03	12,15
Amlexanox	Anti-inflammatory Immunomodulator	-11,48	6,07
Fulvestrant	Antineoplastic	-11,92	37,59
(+) -Levobunolol hydrochloride	Antiglaucoma	-11,94	12,79
Morantel tartrate	Antihelmintic	-11,95	5,28
Fludarabine	Antineoplastic	-12	44,86
Phentolamine hydrochloride	Antihypertensive vasodilator	-12,39	33,97
Estradiol Valerate	Contraceptive	-12,79	39,05
Didanosine	Antiviral	-12,87	35,22
Rosiglitazone Hydrochloride	Antidiabetic	-13,02	22,84
Scopolamine hydrochloride	Antiemetic	-13,14	7,33
Lorglumide sodium salt	Antiulcer	-13,48	18,92
Etanidazole	Antineoplastic chemosensitizer	-13,95	5,37
Fenbufen	Analgesic anti-inflammatory antipytetic	-14,14	43,62
Hexetidine	Antifungal antiseptic	-14,41	3,16
Sulfaguanidine	Antibacterial	-14,59	-8,92
Pentobarbital	Anesthetic hypnotic sedative	-14,62	2,31
Enalapril maleate	Antihypertensive	-14,88	12,35
Dienestrol		-15,46	11,33
Nifedipine	Antianginal antihypertensive vasodilator	-15,96	20,06
Bacitracin	Antibacterial	-566,7	95,46
Gemifloxacin mesylate	Antibacterial	56,18	88,42
Lynestrenol	Contraceptive	-16,03	49,14
Mepivacaine hydrochloride	Local anesthetic	-16,23	15,16
Nomifensine maleate	Antidepressant	-16,29	54,22
Moroxidine hydrochloride	Antiviral	-16,63	33,73
Liranaftate	Antifungal	-17,29	-3,87
Prochlorperazine dimaleate	Antiemetic Antiphychotic	-17,54	31,07
Cladribine	Antineoplastic	-190,03	56,17
Iopromide	Contrastant	-17,55	11,66
Promethazine hydrochloride	Antihistaminic Sedative	-17,87	2,94
Iopamidol	Contrastant	-17,94	13,36
Fluphenazine dihydrochloride	Antipsychotic	-18	22,94
Azilsartan kamedoxomil	Antihypertensive	-18,15	52,25
Bemegride	CNS Stimulant	-18,76	-4,01
Clofilium tosylate	Antirrhythmic	-19,04	1,63

Name	Therapeutic effect	% GraRS inhibition	% Growth inhibition
Thonzonium bromide	Antiseptic	-1906,98	97,79
Deoxycorticosterone	Anti-inflammatory	-19,28	15,63
Meprylcaine hydrochloride	Local anesthetic	-19,37	18,81
Iobenguane sulfate	Antineoplastic	-19,44	10,03
Danazol	Anabolic antigonadotropin	-19,49	48,35
Crotamiton	Antipruritic	-20,88	5,88
Niacin	Antilipemic Vasodilator	-21,64	0,13
Canrenone	Diuretic	-21,66	-2,78
Zalcitabine	Antiviral	-21,74	17,05
Lamivudine	Antiviral	-184,31	57,07
Simvastatin	Antilipemic	-21,83	21,03
Ambroxol hydrochloride	Expectorant Mucolytic	-21,95	47,43
Anastrozole	Antineoplastic	-22,14	9,61
Xylometazoline hydrochloride	Nasal decongestant vasoconstrictor	-22,18	42,41
Pyrantel tartrate	Antihelmintic	-23,18	26,85
Benzthiazide	Antihypertensive Diuretic	-23,29	16,11
Piperacetazine	Antipsychotic	-23,5	4,93
Loperamide hydrochloride	Antidiarrheal	-24,09	46,02
Pergolide mesylate	Antiparkinsonian	-24,87	36,71
Sulfadimethoxine	Antibacterial	-24,87	10,97
Dehydroisoandosterone 3-acetate		-24,89	24,58
Cefaclor hydrate	Antibacterial	-25,06	20,33
Isoxsuprine hydrochloride	Vasodilator	-25,12	15,33
Algestone acetophenide	Contraceptive Anti-inflammatory	-25,44	16,35
Mephentermine hemisulfate	Antihypotensive Vasoconstrictor	-25,67	-0,93
Nevirapine	Antiviral	-25,98	2,05
Idoxuridine	Antiviral	-26	28,24
Paromomycin sulfate	Antiamebic Antibacterial	-26,04	38,02
Sotalol hydrochloride	Antianginal Antiarrhythmic Antihypertenisve	-26,15	0,64
Dosulepin hydrochloride	Antidepressant CNS stimulant	-26,53	6,63
Sulfadoxine	Antibacterial	-26,59	23,44
Azaperone	Antipsychotic Sedative	-26,7	11,73
Eprosartan mesylate	Antihypertenisve	-27,69	17,46
Raloxifene hydrochloride		-175,94	55,81
Bupropion hydrochloride	Antidepressant	-27,97	17,82
Entacapone	Antiparkinsonian	-28,06	10,82
Enilconazole	Antifungal	-28,16	7,65
Procarbazine hydrochloride	Antineoplastic	-28,47	14,3
Buspirone hydrochloride		-28,49	8,38
Methacycline hydrochloride	Antibacterial	91,59	88,74
Floxuridine	Antineoplastic Antiviral	119	73,49
Amiprilose hydrochloride	Immunomodulator	-28,69	20,28

Name	Therapeutic effect	% GraRS inhibition	% Growth inhibition
Propantheline bromide	Antispastic	-28,71	15,62
Indatraline hydrochloride	Antidepressant	-28,82	9,43
Zolmitriptan		-29,48	16,44
Fentiazac	Anti-inflammatory	-29,49	28,52
Sulfamethoxazole	Antibacterial	-29,88	50,57
Carbetapentane citrate	Antispastic Antitussive Local anesthetic	-29,89	35,12
Trimebutine	Antispastic	-30,3	8,05
Dimethadione	Anticonvulsant	-31,02	14,78
Reserpine	Antipsychotic	-31,21	25,06
Dolasetron mesilate	Antiemetic	-31,24	4,27
(R)-Propranolol hydrochloride	Antianginal Antiarrhythmic Antihyperensive	-31,26	20,01
Balsalazide Sodium	Anti-inflammatory	-31,32	13,93
Nelfinavir mesylate	Antiviral Antineoplastic	-31,57	8,27
Darifenacin hydrobromide		-31,57	2,75
Meclofenamic acid sodium salt monohydrate	Anti-inflammatory antipytetic	-31,62	37,53
Pepstatin A	Antiviral	-31,76	18,42
Decamethonium bromide	Muscle relaxant	-31,98	3,55
Nicotinamide		-32,25	12,17
Bezafibrate	Antilipemic Hypocholesterolemic Lipid-lowering	-32,54	34,48
Tolmetin sodium salt dihydrate	Anti-inflammatory	-33,03	29,83
Ciprofibrate	Hypocholesterolemic	-33,08	26,47
Lofepramine	Antidepressant Anxiolytic Sedative	-33,56	13,79
Ioversol	Contrastant	-33,78	13,59
Flurbiprofen	Analgesic Anti-inflammatory	-34,07	36,26
Delavirdine		-34,25	9,57
Althiazide	Antihypertensive	-34,36	3,74
Metolazone	Antihypertensive diuretic	-34,4	49,31
Nicorandil	Antianginal vasodilator	-34,45	50,28
Alfuzosin hydrochloride	Vasodilator	-34,51	8,73
Ranolazine	Antianginal	-34,64	18,65
Mesalamine	Anti-inflammatory	-35,03	17,32
(R)-(+)-Atenolol	Antianginal Antiarrhythmic Antihypertenisve	-35,64	15,89
Antipyrine	Analgesic anti-inflammatory antipyretic	-35,85	19,66
Benzylpenicillin sodium	Antibacterial	-161,88	57,51
Hydroflumethiazide	antihypertensive diuretic	-36,07	34,12
Sertaconazole nitrate	Antibacterial Antifungal	-36,3	28,45
Articaine hydrochloride	Anesthetic	-36,58	6,06
Clidinium bromide	Antispastic	-36,79	21,1
Baclofen (R,S)	Antispastic, muscle relaxant alcohol addiction treatment	-36,9	31,34
Megestrol acetate	Antineoplastic Contraceptive	-37,15	11,31

Name	Therapeutic effect	% GraRS inhibition	% Growth inhibition
Sulfanilamide	Antibacterial	-37,2	1,39
Indoprofen	Analgesic Anti-inflammatory	-37,21	18,34
Donepezil hydrochloride	Anti-Alzheimer Antipsychotic CNS Stimulant	-37,59	20,14
Serotonin hydrochloride	CNS stimulant	-38,31	3,06
Flucloxacillin sodium	Antibacterial	-873,24	96,33
Norethynodrel	Contraceptive	-38,4	25,25
Mecamylamine hydrochloride	Antihypertensive	-38,43	18,72
Carbimazole	Antihyperthyroid	-158,16	60,16
Ethionamide	Antibacterial	-38,71	20,1
Droperidol	Antipsychotic	-39,45	30,66
Alprenolol hydrochloride	Antianginal antiarrhythmic antihypertensive	-39,6	20,27
Colistin sulfate	Antibacterial	-40,03	7,24
Rivastigmine		-40,28	18,34
Deflazacort	Anti-inflammatory Immunosuppressant	-40,63	30,15
Chicago sky blue 6B		-40,83	12,46
Mafenide hydrochloride	Antibacterial antiseptic	-41,09	25,49
Dorzolamide hydrochloride	Antiglaucoma Antihypertensive	-41,49	23,9
Clioquinol	Antiamebic Antifungal Antiseptic	-41,7	12,03
Vecuronium bromide	Muscle relaxant	-42,01	38,56
Levofloxacin	Antibacterial	-18,19	84,78
Rebamipide	Antiulcer	-42,04	-2,32
Mianserine hydrochloride	Antidepressant anxiolytic	-42,11	54,9
Fenoldopam	Antihypertenisve Vasodilator	-42,15	16,48
Nylidrin	Vasodilator	-42,16	19,62
Procaine hydrochloride	Local anesthetic	-42,26	24,61
Saquinavir mesylate	Antiviral	-42,73	18,75
Fluocinolone acetonide	Anti-inflammatory	-42,78	29,68
Vatalanib	Antineoplastic	-42,79	13,83
Itraconazole	Antifungal	-43,49	7,03
Dapsone	Antibacterial antimalarial	-43,51	27,04
Toremifene	Antineoplastic	-43,53	15,8
Topiramate	Anticonvulsant Antimigraine	-43,96	24,78
Tribenoside		-43,97	27,84
Butoconazole nitrate	Antibacterial antifungal	-44,13	36,77
Phenethicillin potassium salt	Antibacterial	-44,54	26,1
Histamine dihydrochloride	Antineoplastic Analgesic	-44,94	17,03
Nomegestrol acetate	Contraceptive	-45,08	15,32
Isopyrin hydrochloride	Analgesic Anti-inflammatory antipyretic	-45,41	0,1
Pancuronium bromide	Muscle relaxant	-45,42	8,38
Glibenclamide	Antidiabetic	-45,58	19,29
Acetylsalicylsalicylic acid	Analgesic anticoagulant anti-inflammatory	-45,9	52,71

Name	Therapeutic effect	% GraRS inhibition	% Growth inhibition
S(-)Eticlopride hydrochloride		-46,05	19,06
Fenbendazole	Antihelmintic	-46,46	42,38
Dropropizine (R,S)	Antitussive	-46,68	23,53
Nialamide	Antidepressant	-46,77	23,53
Oxalamine citrate salt	Anti-inflammatory Antispastic Antitussive	-47,48	24,45
Eserine hemisulfate salt	Antiglaucoma	-47,65	8,28
Benperidol	Antipsychotic	-47,77	4,37
(-)-Isoproterenol hydrochloride	Bronchodilator Vasodilator	-48,08	25,75
Pentylenetetrazole	Analeptic CNS stimulant	-48,36	29,53
Ethoxzolamide	Antiglaucoma Antiulcer Diuretic	-48,82	11,04
Nifenazone	Analgesic anti-inflammatory antipytetic	-48,87	41,43
Pheniramine maleate	Antihistaminic antitussive sedative	-49,13	21,16
Neostigmine bromide	Anti-fatigue	-49,28	41,43
Metyrapone		-49,42	16,42
Amphotericin B	Antibacterial antifungal	-49,42	17,85
Hexestrol	Antineoplastic	-49,77	34,27
Clocortolone pivalate	Anti-inflammatory	-49,88	15,29
Nefazodone hydrochloride	Antidepressant	-50,74	37,07
D-cycloserine	Antibacterial	-50,91	29,21
Ioxaglic acid	Contrastant	-50,93	27,16
Cilnidipine	Antihypertensive	-51,4	47,94
Procyclidine hydrochloride	Antiparkinsonian Muscle relaxant	-51,89	31,5
Dicyclomine hydrochloride	Antispastic	-52,29	30,98
Ethaverine hydrochloride	Antispastic	-52,31	27,27
Avermectin B1	Antihelmintic	-52,87	52,15
Diclofenac sodium	Anti-inflammatory	-138,18	60,5
Ketoprofen	Analgesic anti-inflammatory antipytetic	-53,21	36,49
Bicalutamide	Antineoplastic	-53,59	22,13
Naftifine hydrochloride	Antifungal	-53,66	37,86
Mefexamide hydrochloride	CNS Stimulant	-53,78	48,05
Tazarotene	Antipsoriatic antiacne	-53,78	12,37
N-Acetyl-DL-homocysteine Thiolactone	Expectorant	-53,91	-2,78
Idazoxan hydrochloride	Antiparkinsonian Antipsychotic	-54	19,96
Itopride		-54,1	10,52
Ethambutol dihydrochloride	Antibacterial	-54,17	33,98
Loxapine succinate	Antipsychotic anxiolytic	-54,31	42,15
Ronidazole	Antibacterial Antiprotozoal Antitrichomonal	-54,42	26,93
Celecoxib	Anti-inflammatory	-55,09	35,82
Estrone		-55,22	27,77
Bethanechol chloride		-55,47	36,84
Iopanoic acid	Contrastant	-55,73	47,98

Name	Therapeutic effect	% GraRS inhibition	% Growth inhibition
Panthenol (D)	Anti-alopecia	-55,74	26,13
Nafronyl oxalate	Anti-ischemic Antispastic Vasodilator	-55,77	41,61
Mexiletine hydrochloride	Antirrhythmic local anesthetic	-55,78	15,94
Levalbuterol hydrochloride	Antiasthmatic Bronchodilator	-56,2	34
Acarbose	Antidiabetic	-56,2	13,37
Glutethimide, para-amino	Antineoplastic	-56,24	13
Atorvastatin		-56,45	26,4
Enalaprilat dihydrate	Antihypertenisve	-56,69	17,43
Iodixanol	Contrastant	-56,77	26,9
Sarafloxacin	Antibacterial	50,98	78,47
Rifabutin	Antibacterial	-263,66	94,54
Apramycin	Antibacterial	-57,08	24,34
4-aminosalicylic acid	Antibacterial Antifungal	-57,38	32,03
Estramustine	Antineoplastic	-57,75	28,39
Perindopril	Antihypertenisve	-57,86	35,66
Quinapril hydrochloride	Antihypertensive	-58,34	37,86
Bufexamac	Analgesic anti-inflammatory antipyretic	-58,36	-0,36
Primaquine diphosphate	Antimalarial	-58,57	19,87
Mirtazapine	Antidepressant	-58,58	18,26
Benzoxiquine	Antiseptic	-58,6	26,12
Acetaminophen	Analgesic Antipyretic	-58,62	30,78
Nadifloxacin	Antibacterial	19,1	91,49
Fluvoxamine maleate	Antidepressant CNS Stimulant	-58,62	20,82
Viomycin sulfate	Antibacterial	-58,64	21,3
Anthralin	Antipsoriatic	-58,7	35,17
Mevastatin	Hypocholesterolemic	-58,74	7,12
Triamterene	antihypertensive diuretic	-58,8	33,33
Atropine sulfate monohydrate	Antispastic mydriatic	-58,8	10,64
Stavudine	Antiviral	-59,02	42,59
Cefepime hydrochloride	Antibacterial	-627,3	96,49
Rifaximin	Antibacterial	-73,94	94,16
Fluvastatin sodium salt	Antilipemic	-59,17	21,35
Selegiline hydrochloride	Antiparkinsonian	-59,32	8,09
Valdecoxib	Antiarthritic Anti-inflammatory	-59,55	23,53
Doxycycline hydrochloride	Antibacterial	103,15	89,58
Carbadox	Antibacterial	-128,95	68,09
Fleroxacin	Antibacterial	71,83	75,89
Clavulanate potassium salt	Antibacterial	-182,94	69,76
Nalmefene hydrochloride		-59,61	29,23
Ethopropazine hydrochloride	Antiparkinsonian	-60,17	37,18
Perospirone	Antipsychotic	-60,17	23,07
Pyridostigmine iodide		-60,19	7,04

Name	Therapeutic effect	% GraRS inhibition	% Growth inhibition
Levocabastine hydrochloride	Antihistaminic	-60,46	13,11
Adamantamine fumarate	Antiviral	-60,47	14,13
Homatropine hydrobromide (R,S)	Antispastic mydriatic	-60,49	23,02
Iocetamic acid	Contrastant	-60,5	31,59
Antipyrine, 4-hydroxy		-60,54	29,84
Acebutolol hydrochloride	Antianginal antiarrhythmic antihypertensive	-60,61	21,49
Benoxinate hydrochloride	Local anesthetic	-61,1	17,15
Urosiol		-61,13	18,36
Imatinib	Antineoplastic	-61,16	17,69
Oxethazaine	Local anesthetic	-61,6	27,72
Spironolactone	Diuretic	-62,18	47,47
Diphenhydramine hydrochloride	Antiemetic antihitaminic antitussive	-62,3	26,15
Cefdinir	Antibacterial	-543,28	96,98
Phenothiazine	Antipsychotic Antiemetic Antimigraine	-62,33	23,09
Nabumetone	Analgesic Anti-inflammatory	-62,68	15,39
Formoterol fumarate	Antiasthmatic	-62,82	21,37
Montelukast	Antiasthmatic	-62,85	41,46
Butacaine	Anesthetic	-62,86	36,75
Etilefrine hydrochloride	Vasoconstrictor	-62,93	29,33
Nicardipine hydrochloride	Antianginal Antihypertensive	-62,98	36,03
Cyproterone acetate	Antineoplastic Contraceptive	-63,04	31,66
Flucytosine	Antifungal	-63,07	24,9
Ceftibuten	Antibacterial	-63,6	43,19
Captopril	Antihypertensive vasodilator	-63,65	32,62
Triclosan	Antibacterial Antifungal Antiseptic	66,77	92,54
Enoxacin	Antibacterial	59,74	75,6
Prothionamide	Antibacterial	-63,98	23,13
Latanoprost	Antiglaucoma	-64,27	35,04
Pramipexole dihydrochloride	Antiparkinsonian	-64,32	24,22
Flumethasone pivalate	Anti-inflammatory	-64,7	21,65
Telmisartan	Antihypertensive	-122,3	56,98
Trapidil	Vasodilator	-64,79	25,38
Ticarcillin sodium	Antibacterial	-65,01	39,96
Gliclazide	Anticoagulant antidiabetic	-65,1	20,94
2-Aminobenzenesulfonamide	Diuretic	-65,24	26,23
Sparfloxacin	Antibacterial	93,31	77,7
Palonosetron hydrochloride	Antiemetic	-65,67	24,35
Clarithromycin	Antibacterial	-78,27	78,85
Trimeprazine tartrate	Antihistaminic Antipruritic Sedative	-65,67	36,24
Ezetimibe	Hypocholesterolemic	-65,89	30,12
Sertraline	Antidepressant CNS Stimulant	-65,92	27,77
Pantoprazole sodium	Antiulcer	-65,93	25,01

Name	Therapeutic effect	% GraRS inhibition	% Growth inhibition
Azithromycin	Antibacterial	47,23	76,8
Pioglitazone		-65,94	49,33
Iodipamide	Contrastant	-66	22,47
Trichlormethiazide	Antihypertensive Diuretic	-66,22	34,42
Bromperidol	Antipsychotic	-66,5	24,11
Chloramphenicol	Antibacterial	-66,59	31,82
Ritonavir	Antiviral	-66,76	23,43
Oxymetholone	Anabolic	-66,77	30,74
Gatifloxacin	Antibacterial	46,56	85,07
Prazosin hydrochloride	Antihyperensive	-66,79	41,03
Gemcitabine	Antineoplastic	16,91	77,03
Equilin		-67,5	31,04
Anagrelide	Thrombolytic	-67,62	27,36
Amrinone		-67,72	23,98
Bucladesine sodium salt		-67,95	27,44
Sulfacetamide sodic hydrate	Antibacterial Antipsoriatic	-68,19	31,39
Chlorpromazine hydrochloride	Antiemetic antihypertensive antipsychotic	-68,2	21,73
Hexachlorophene	Antiseptic	-89,63	96,86
Ampicillin trihydrate	Antibacterial	-68,37	51,2
Moxifloxacin	Antibacterial	81,62	85,18
Pralidoxime chloride		-68,46	32,36
Rufloxacin	Antibacterial	185,42	78,28
Methocarbamol	Analgesic muscle relaxant	-68,77	37,24
Doxylamine succinate	Anti-anorectic antiemetic antihitaminic	-69,42	31,34
Nitrendipine	Antihypertenisve	-69,5	20,56
Pyrvinium pamoate		-69,57	47,75
Betazole hydrochloride	Diagnostic	-69,68	34,62
Ibudilast	Anti-inflammatory	-69,76	26,66
Fosfosal	Analgesic	-70,02	27,64
Ampiroxicam	Anti-inflammatory Analgesic	-70,56	20,67
Pinacidil	Antihypertensive vasodilator	-70,78	23,63
Guanabenz acetate	Antihypertensive	-70,81	49,39
Chlormadinone acetate	Antineoplastic	-70,86	22,71
Ifenprodil tartrate	Vasodilator	-71,29	-2,74
Flurandrenolide	Anti-inflammatory antipruritic	-71,6	7,16
Enrofloxacin	Antibacterial	57,64	79,49
Aceclofenac	Analgesic anti-inflammatory	-71,63	47,77
Pindolol	Antianginal antiarrhythmic antiglaucoma	-71,65	16,73
Piracetam	CNS stimulant	-71,74	32,16
Proparacaine hydrochloride	Anesthetic	-71,74	16,25
Butalbital	Hypnotic sedative	-71,9	30,64
Capecitabine	Antineoplastic	-72,51	36,58

Name	Therapeutic effect	% GraRS inhibition	% Growth inhibition
Theophylline monohydrate	Bronchodilator CNS Stimulant Diuretic	-72,58	17,5
Carvedilol	Antihypertensive	-73,15	38,7
Tolcapone	Antiparkinsonian	-153,68	80,96
Naphazoline hydrochloride	Nasal Decongestant Vasoconstrictor	-73,21	31,21
Vardenafil	Erectile dysfunction treatment	-73,5	30,48
Cromolyn disodium salt	Antiasthmatic Anti-inflammatory	-73,73	36,78
Pirenzepine dihydrochloride	Antiulcer	-73,78	53,32
Methiazole	Antihelmintic	-74,31	35,26
Astemizole	Antihistaminic	-74,53	45,44
Nitazoxanide	Antiprotozoal	-156,12	67,85
Levodopa	Antiparkinsonian	-74,56	32,69
Propoxycaine hydrochloride	Anesthetic	-74,69	35,08
Hexamethonium dibromide dihydrate	Antihypertensive	-74,88	38,01
Pipemidic acid	Antibacterial	-75	31,5
Pidotimod	Immunostimulant	-75,19	30,25
Retinoic acid	Keratolytic	-75,34	31,67
Meticrane	antihypertensive diuretic	-75,48	30,78
Diflunisal	Analgesic anti-inflammatory antipyretic	-75,59	36,34
Formestane	Antineoplastic	-75,81	37,55
Salmeterol	Bronchodilator	-76,08	35,89
Furaltadone hydrochloride	Antibacterial	-76,32	42,27
Benfluorex	Anorectic Antidiabetic CNS Stimulant	-76,34	48,19
(-)-Eseroline fumarate salt	Analgesic	-76,36	38,77
Tolazoline hydrochloride	Vasodilator	-76,63	29,47
Sulfaphenazole	Antibacterial	-76,71	33,25
Betaxolol hydrochloride	Antiglaucoma Antihypertensive	-77,05	33,04
Cefuroxime axetil	Antibacterial	-292,99	81,47
Doxazosin mesylate	Antihypertensive	-77,09	32,45
Thiocolchicoside	Antispastic muscle relaxant	-77,1	25,35
Deferoxamine mesylate	Chelating	-77,6	11,92
(-) -Levobunolol hydrochloride	Antiglaucoma	-77,83	32,28
Oxfendazol		-77,96	22,73
Dofetilide	Antiarrhythmic	-77,98	28,67
Isradipine	Antianginal Antihypertenisve	-78,27	28,94
Besifloxacin hydrochloride	Antibacterial	3,54	93,13
Ritodrine hydrochloride	Tocolytic	-78,27	33,08
GBR 12909 dihydrochloride	Antidepressant	-78,44	40,62
Benzydamine hydrochloride	Analgesic anti-inflammatory antipyretic	-78,45	21,55
Naltrexone hydrochloride dihydrate	Analgesic	-78,65	44,74
Methotrimeprazine maleat salt	Analgesic antiemetic sedative	-78,73	24,75
Moxisylyte hydrochoride	Erectile dysfunction treatment vasodilator	-78,87	32,03
Tolbutamide	Antidiabetic	-79,31	42,04

Name	Therapeutic effect	% GraRS inhibition	% Growth inhibition
Methicillin sodium		-108,81	61,23
Ethacrynic acid	Diuretic	-79,47	36,71
Minoxidil	Anti-alopecia antihypertensive vasodilator	-79,54	23,87
Trimethadione	Anticonvulsant antiepileptic	-79,69	26,49
Viloxazine hydrochloride	Antidepressant	-80,39	31,41
Beclomethasone dipropionate	Anti-inflammatory	-80,42	33,68
Methylhydantoin-5-(L)	Anticonvulsant	-80,74	32,02
Nandrolone	Antianemic	-80,83	25,62
Isotretinoin	Keratolytic	-80,84	39,59
Tolterodine tartrate	Muscle relaxant	-81,01	29,02
Isoxicam	Analgesic anti-inflammatory antipyretic	-81,11	34,02
Methylhydantoin-5-(D)		-81,23	39,9
Ramipril	Antihypertensive	-81,32	39,43
Nadolol	Antianginal Antihypertensive	-81,57	28,71
Darunavir		-81,92	27,33
Fenspiride hydrochloride	Antitussive bronchodilator	-82,49	35,5
Lamotrigine	Anticonvulsant	-82,56	34,85
Eszopiclone	Hypnotic	-82,74	30,69
Biperiden hydrochloride	Antiparkinsonian	-82,74	27,8
Amiodarone hydrochloride	Antianginal Antiarrhythmic	-82,98	39,24
Isosorbide mononitrate	Antianginal	-83,08	38,07
Racepinephrine hydrochloride	Bronchodilator Vasoconstrictor	-83,27	40,22
Hemicholinium bromide	Curarizing	-83,5	22,9
Cyclopenthiazide	Antihypertensive Diuretic	-83,52	30,86
Ganciclovir	Antiviral	-83,68	35,7
Theobromine	Bronchodilator Diiuretic	-83,79	18,3
6-Furfurylaminopurine		-83,79	30,55
Gabapentin	Anticonvulsant	-83,82	32,52
Valacyclovir hydrochloride	Antiviral	-83,86	29,66
Felbamate	Antiepileptic	-83,9	25,32
Acitretin	Antipsoriatic	-83,94	37,88
Methantheline bromide	Antispastic	-84,06	36,12
Aliskiren hemifumarate	Antihypertensive	-84,08	51,77
Abacavir Sulfate	Antiviral	-84,24	25,36
Dehydrocholic acid	Choleretic	-84,61	43,92
Sulfadiazine	Antibacterial	-84,96	35,72
Aminophylline	Bronchodilator CNS Stimulant Diuretic	-85,43	33,71
Sulfamonomethoxine	Antibacterial	-85,49	34,48
Lacosamide	Analgesic	-85,65	42,45
Azacyclonol	Antipychotic	-85,74	49,8
Trifluridine	Antiviral	-85,96	40,26
Tranylcypromine hydrochloride	Antidepressant	-85,97	39,35

Name	Therapeutic effect	% GraRS inhibition	% Growth inhibition
Oxaprozin	Analgesic Anti-inflammatory	-86,02	38,05
Chlorpropamide	Antidiabetic	-86,19	30,69
Aprepitant	Antiemetic	-86,3	25,16
Etodolac	Analgesic anti-inflammatory antiplatelet	-86,55	7,19
Remoxipride Hydrochloride	Antipsychotic	-86,75	33,14
(S)-Naproxen	Analgesic anti-inflammatory antipyretic	-86,89	27,44
Sibutramine hydrochloride		-86,95	27,39
Altrenogest	Progestogen	-87,03	26,53
Minaprine dihydrochloride	Anti-Alzheimer antidepressant	-87,05	23,96
Milrinone	Vasodilator	-87,12	36,15
Guanfacine hydrochloride	Antihypertensive	-87,5	27,84
Niclosamide	Antihelmintic	116,07	90,79
Raclopride		-87,61	19,36
Lidocaine hydrochloride	Antiarrhythmic local anesthetic	-87,63	28,02
Camptothecine (S,+)	Antineoplastic	-87,69	42,31
Isometheptene mucate	Antimigraine Vasoconstrictor	-87,95	32,75
Timolol maleate salt	Antianginal Antiarrhythmic Antiglaucoma	-87,98	34,73
Heptaminol hydrochloride	Analeptic Positive inotropic Vasodilator	-88,19	31,1
Zoledronic acid hydrate	Antiosteoporosis	-88,21	27,42
R(-) Apomorphine hydrochloride hemihydrate	Antiparkinsonian emetic	-88,26	45,85
Dexfenfluramine hydrochloride	Anorectic	-88,3	21,82
Pentetic acid	Chelating Radioprotectant	-88,51	36,08
Ciclesonide		-89,18	34,19
Nimesulide	Anti-inflammatory	-89,45	41,47
Xamoterol hemifumarate		-89,65	31,65
Procainamide hydrochloride	Antiarrhythmic Local anesthetic Vasodilator	-89,87	38,31
Avobenzone	Cytoprotectant	-89,94	22,53
Alcuronium chloride	Muscle relaxant	-90,3	21,55
Flutamide	Antineoplastic	-90,34	35,22
Benzonatate	Antitussive Local anesthetic	-90,44	36,13
Mebendazole	Antihelmintic	-90,46	45,43
Piperidolate hydrochloride	Antispastic	-90,49	30,25
Mupirocin		697,49	90,55
Nocodazole	Antineoplastic	-90,59	38,69
Amcinonide	Anti-inflammatory	-90,6	37,23
Pregabalin	Anticonvulsant Anxiolytic	-91	26,29
Meclozine dihydrochloride	Antiemetic antihistaminic sedative	-91,05	40,88
Urapidil hydrochloride	Antihypertenisve Vasodilator	-91,15	32,12
(+)-Isoproterenol (+)-bitartrate salt	Antiasthmatic Bronchodilator Vasodilator	-91,3	37,05
Ethamivan	Analeptic CNS stimulant	-91,36	24,39
Alverine citrate salt	Antispastic	-91,4	43,1

Name	Therapeutic effect	% GraRS inhibition	% Growth inhibition
Lansoprazole	Antiulcer	-91,51	40,13
Pentamidine isethionate	Antifungal antiparasitic antiprotozoal	-91,52	21,99
Nateglinide	Antidiabetic	-91,59	33,72
Luteolin	Expectorant	-91,67	33,09
Tropicamide	Mydriatic	-91,82	35,29
Cyclobenzaprine hydrochloride	Muscle relaxant	-92,37	48,86
Chenodiol	Cholagogue Choleretic	-92,42	31,17
Azatadine maleate	Antihistaminic	-92,53	27,1
Pivampicillin	Antibacterial	-92,59	35,73
Gefitinib	Antineoplastic	-92,64	1,49
Amyleine hydrochloride	Local anesthetic	-92,66	36,21
Lovastatin	Hypocholesterolemic	-92,78	28,42
Ceftazidime pentahydrate	Antibacterial	-92,88	34,64
Proguanil hydrochloride	Antimalarial	-93,07	37,02
Thiamphenicol	Antibacterial	-93,09	36,43
Nimodipine	Vasodilator	-93,27	34,19
Homoveratrylamine	Antihypertenisve	-93,71	30,75
Etifenin	Chemosensitizer	-93,83	27,42
Prenylamine lactate	Antianginal anxiolytic vasodilator	-94,3	30,27
Zonisamide	Anticonvulsant	-94,41	23,1
Domperidone	Antiemetic	-94,59	21,74
Benserazide hydrochloride	Antiparkinsonian	-94,79	37,55
Parbendazole		-94,9	49,73
Irbesartan	Antihypertensive	-95,26	37,24
Terbinafine	Antifungal	-95,4	36,77
Nelarabine		-95,62	38,72
Actarit	Anti-inflammatory Immunomodulator	-95,89	28,16
Nisoxetine hydrochloride	Antidepressant	-95,97	34,42
Epirizole	Analgesic anti-inflammatory antipyretic	-96	37,42
Diltiazem hydrochloride	antianginal antiarrhythmic antihypertensive	-96,14	39,91
Methylatropine nitrate	Antispastic Mydriatic	-96,35	34,09
Dioxybenzone		-96,59	32,33
Paroxetine Hydrochloride	Antidepressant CNS Stimulant	-96,65	39,8
Olopatadine hydrochloride	Antihistaminic	-96,78	45,99
Salbutamol	Bronchodilator tocodytic	-97,15	40,99
Stanozolol		-97,38	45,23
Levamisole hydrochloride	Antihelmintic immunomodulator	-97,76	33,22
Metoclopramide monohydrochloride	Antiemetic	-97,78	38,72
Ciclopirox ethanolamine	Antibacterial antifungal	-97,82	31,43
Picotamide monohydrate	Anticoagulant antiplatelet thrombolytic	-97,92	52,93
Vorinostat	Antineoplastic	-97,99	38,43
Clorsulon	Antihelmintic	-98,09	30,75

Name	Therapeutic effect	% GraRS inhibition	% Growth inhibition
Benidipine hydrochloride	Antihypertenisve	-98,21	29,82
Azlocillin sodium salt	Antibacterial	-98,29	52,04
Naftopidil dihydrochloride	Antihypertensive	-98,74	29,35
Fluocinonide	Anti-inflammatory	-98,74	26,58
Tolnaftate	Antifungal	-99,07	22,7
Diprophylline	Analeptic antispastic bronchodilator	-99,35	40,88
Lodoxamide	Antihistaminic	-99,4	33,28
Ziprasidone Hydrochloride	Antipsychotic	-99,56	24,78
Propafenone hydrochloride	Antiarrhythmic	-99,76	26,43
Cimetidine	Antiulcer	-99,96	36,06
Hymecromone	Muscle relaxant	-100,21	36,05
Ciprofloxacin hydrochloride monohydrate	Antibacterial antiprotozoal	318,88	75,9
Oxolinic acid	Antibacterial	-100,69	43,75
Oxprenolol hydrochloride	Antianginal Antiarrhythmic Antihyperensive	-100,83	35,25
Imiquimod	Antiviral	-100,93	36,27
Yohimbine hydrochloride	Erectile dysfunction treatment vasodilator	-100,93	29,93
Sulfathiazole	Antibacterial	-101,05	34,61
Famotidine	Antiulcer	-101,11	44,08
Flunisolide	Anti-inflammatory	-101,16	4,99
Fexofenadine hydrochloride	Antihistaminic	-101,24	43,72
Estriol		-102,31	41,82
Fluticasone propionate	Anti-inflammatory Vasodilator	-102,34	39,35
Isocarboxazid	Antidepressant	-102,59	29,68
Iproniazide phosphate	Antidepressant antihypertensive	-102,6	46,73
Diflorasone Diacetate	Anti-inflammatory antipruritic antipsoriatic	-102,95	39,56
Isoconazole	Antibacterial antifungal	157,84	71,03
Terfenadine	Antihistaminic antipruritic	-103,02	47,57
Cefotaxime sodium salt	Antibacterial	904,42	93,91
Tetracycline hydrochloride	Antibacterial	507,2	89,84
Fluspirilen	Antipsychotic	-103,03	42,46
Allopurinol		-103,15	32,74
Imidurea	Antifungal	-103,25	40,84
Diloxanide furoate	Antiamebic	-103,3	32,57
Amfepramone hydrochloride		-103,32	48,19
(R)-Duloxetine hydrochloride		-103,38	39,02
Clindamycin hydrochloride	Antibacterial	821,87	91,69
Lymecycline	Antibacterial	-103,49	38,55
Protriptyline hydrochloride	Antidepressant	-103,57	38,31
Norgestimate		-103,62	49,57
Rasagiline	Antiparkinsonian	-103,68	31,41
Flubendazol		-103,69	30,94

Name	Therapeutic effect	% GraRS inhibition	% Growth inhibition
Chlorhexidine	Antibacterial antiseptic	1668,32	97,77
Sertindole	Antipsychotic	-103,79	21,36
Chlortetracycline hydrochloride	Antiamecib antibacterial	512,98	92,51
Tamoxifen citrate	Antineoplastic	63,72	61,04
Clopidogrel	Antiplatelet	-104,24	36,31
Cefoxitin sodium salt	Antibacterial	-83,74	70,78
Dihydrostreptomycin sulfate	Antibacterial	277,27	85,53
Gentamicine sulfate	Antibacterial	440,04	76,9
Erythromycin	Antibacterial anti-inflammatory	723,07	91,66
Chloroxine		349,51	78,59
Phenprobamate	Muscle relaxant Sedative Anticonvulsant	-104,39	25,62
Josamycin	Antibacterial	557,7	93,87
Aminopurine, 6-benzyl		-105,16	38,6
Tiaprofenic acid	Analgesic anti-inflammatory antipyretic	-105,17	30,97
Diatrizoic acid dihydrate	Contrastant	-105,35	32,5
Tulobuterol	Bronchodilator	-105,41	38,81
Clemizole hydrochloride	Antibacterial antifungal antihistaminic	-105,59	51,25
Liothyronine		-105,65	37,42
Pyrimethamine	Antimalarial antiprotozoal	-105,74	42,48
Phenoxybenzamine hydrochloride	Antihypertensive	-106,01	37,37
Methenamine	Antibacterial	-106,04	43,48
Felodipine	Antianginal antihypertensive	-106,4	22,71
Acetohexamide	Antidiabetic	-106,41	32,62
Tolazamide	Antidiabetic	-106,51	21,64
Losartan	Antihypertensive	-107,01	49,19
Ethynodiol diacetate	Contraceptive	-107,42	39,56
Benztropine mesylate	Antiparkinsonian	-107,54	43,26
Pyrazinamide	Antibacterial	-107,58	31,43
Clozapine	Antiparkinsonian Antipsychotic	-107,6	30,24
Tocainide hydrochloride	Anesthetic Antiarrhythmic	-107,7	41,15
Oxandrolone		-107,76	40,12
(S)-propranolol hydrochloride	Antianginal Antiarrhythmic Antihyperensive	-107,94	42,55
Cephalosporanic acid, 7-amino	Antibacterial	-108,02	32,48
(+,-)-Synephrine	Vasoconstrictor	-108,06	36,68
Molindone hydrochloride	Antipsychotic	-108,12	19,77
(S)-(-)-Cycloserine	Antibacterial	-108,83	39,52
Pridinol methanesulfonate salt	Antiparkinsonian	-109,03	28,88
Pivmecillinam hydrochloride	Antibacterial	-109,08	39,11
Docetaxel	Antineoplastic	-109,08	48,65
Phensuximide	Anticonvulsant	-109,72	43,66
Phthalylsulfathiazole	Antibacterial	-109,99	34,43
Monobenzone		-110,36	36,89

Name	Therapeutic effect	% GraRS inhibition	% Growth inhibition
Nilvadipine	Antianginal antihypertensive	-110,36	14,19
Cloxacillin sodium salt	Antibacterial	-3481,68	97,96
Carteolol hydrochloride	Antiglaucoma antihypertensive	-110,36	30,27
Propidium iodide	Antibacterial	-110,43	25,42
Spaglumic acid	Antiallergic Vasodilator	-110,68	47,2
Levopropoxyphene napsylate	Analgesic Antitussive	-110,78	41,3
Meropenem	Antibacterial	-77,35	65,94
Tegaserod maleate	Gastreoprokinetic	-110,93	24,89
Sulfisoxazole	Antibacterial	-110,98	38,48
Phenindione	Anticoagulant	-111,13	36,21
Nifuroxazide	Antibacterial	-111,21	23,26
Bephenium hydroxynaphthoate		-111,71	33,83
Gliquidone	Antidiabetic	-111,74	38,57
Niflumic acid	Analgesic anti-inflammatory antipyretic	-111,81	33,92
Ropivacaine hydrochloride	Anesthetic	-112,36	30,12
Haloperidol	Antiemetic antipsychtotic	-112,51	45,81
Lomerizine hydrochloride	Antimigraine	-112,59	35,98
Amodiaquin dihydrochloride dihydrate	Anti-inflammatory antimalarial	-112,61	28,5
Fosinopril	Antihypertenisve	-112,67	44,87
Posaconazole	Antifungal	-112,7	32,09
Clonixin Lysinate	Analgesic Antifungal	-112,72	46,78
Perphenazine	Antiemetic antipsychotic	-112,76	45,76
Ticlopidine hydrochloride	Anticoagulant antiplatelet	-112,99	36,58
Ambrisentan	Antihypertensive	-113,24	37,48
Phenylbutazone	Anti-inflammatory	-113,26	38,76
Butylparaben	Antifungal	-113,53	30,11
Griseofulvin	Antifungal anti-inflammatory	-114,03	44,62
Phenformin hydrochloride	Antidiabetic	-114,06	41,65
Nilutamide	Antineoplastic	-114,32	32,66
Nystatine	Antifungal	-114,5	43,69
Glipizide	Antidiabetic	-114,93	40,32
Cyproheptadine hydrochloride	Antihistaminic antipruritic sedative	-115,61	49,45
Tripelennamine hydrochloride	Antihistaminic	-116	43,21
Risedronic acid monohydrate	Antiosteoporosis	-116,41	27,68
Adapalene	Keratolytic Anti-inflammatory	-116,43	43,29
Etoricoxib	Analgesic anti-inflammatory	-116,53	32,73
Norfloxacin	Antibacterial	85,65	78,14
Cloperastine hydrochloride	Antitussive	-116,69	25,72
Dopamine hydrochloride	Antihypertensive	-116,73	33,91
Trimetazidine dihydrochloride	Antianginal antischemic vasodilator	-116,78	31,21
Sulfasalazine	Antibacterial anti-inflammatory	-116,96	31,18
Ethoxyquin	Antifungal	-117,13	26,14

Name	Therapeutic effect	% GraRS inhibition	% Growth inhibition
Clobetasol propionate	Anti-inflammatory	-117,17	30,31
Homosalate	Radioprotectant	-117,23	38,83
Moxonidine	Antihypertenisve	-117,33	38,39
Toltrazuril	Anticoccidial	-117,4	44,7
Pentolinium bitartrate	Antihypertensive	-117,49	47,71
Amethopterin (R,S)	Anti-inflammatory Antineoplastic Immunosuppressant	-117,98	47,44
Olmesartan	Antihypertensive	-118,17	40,86
Trimethoprim	Antibacterial antimalarial	-118,56	42,83
Terbutaline hemisulfate	Antiasthmatic Bronchodilator Muscle relaxant	-118,57	8,47
Telenzepine dihydrochloride	Antiulcer	-118,62	32,14
Ofloxacin	Antibacterial	71,59	81,8
Lomefloxacin hydrochloride	Antibacterial	102,61	77,55
N6-methyladenosine	Antineoplastic	-118,68	33,69
Azapropazone	Analgesic Anti-inflammatory	-118,81	35,82
Flumequine	Antibacterial	-118,91	52,63
Propylthiouracil	Antihyperthyroid	-118,96	31,86
Aminacrine	Antiseptic	-118,97	36,7
N-Acetyl-L-leucine	Antivertigo	-118,98	34,65
Ipriflavone	Antiosteoporosis	-119,09	32,83
Mitotane	Antineoplatic	-119,35	41,41
Rizatriptan benzoate	Antimigraine Vasoconstrictor	-119,46	28,48
Felbinac	Analgesic Anti-inflammatory	-119,71	28,67
Halofantrine hydrochloride	Antimalarial	-119,74	45,21
Ketorolac tromethamine	Analgesic Anti-inflammatory Antipyretic	-119,81	34,37
Diclazuril		-119,9	49,72
Digitoxigenin	Cardiotonic	-119,97	35,71
Aceclidine Hydrochloride	Antiglaucoma	-120,09	36,35
Fluoxetine hydrochloride	Antidepressant	-120,33	30,18
Guaiacol	Expectorant	-120,38	38,02
Alosetron hydrochloride	Antidiarrheal	-120,4	39
Flunixin meglumine	Analgesic Anti-inflammatory antipyretic	-120,55	40,91
Adenosine 5'-monophosphate monohydrate	Antiarrhythmic	-120,6	42,89
Debrisoquin sulfate	Antihypertensive	-120,95	29,24
Terconazole	Antifungal	-120,95	28,43
Molsidomine	Antianginal anticoagulant antiplatelet	-121,21	31,94
Naloxone hydrochloride	Opioate antidote	-121,46	44,9
Tomoxetine hydrochloride		-121,53	27,37
Trolox	Antioxidant	-121,9	32,46
Norcyclobenzaprine	Antiulcer	-122,49	36,27
Cilostazol	Anticoagulant	-122,51	27,06

Name	Therapeutic effect	% GraRS inhibition	% Growth inhibition
Ethinylestradiol	Contraceptive	-122,84	36,57
Cyclopentolate hydrochloride		-122,89	48,17
Cefadroxil	Antibacterial	-122,95	43,38
Modafinil	CNS stimulant	-123,16	34,62
Olanzapine	Antipsychotic	-123,22	40,68
Trazodone hydrochloride	Antidepressant	-123,48	24,86
Acenocoumarol	anticoagulant	-123,53	37,99
Bepridil hydrochloride	Antianginal Antiarrhythmic Antihypotensive	-123,74	44,03
Meptazinol hydrochloride	Analgesic	-123,91	32,49
Tiabendazole	Antifungal Antihelmintic antiparasitic	-123,96	37,85
Bifonazole	Antifungal	-124,19	49
Chloroquine diphosphate	Anti-inflammatory antimalarial antiprotozoal	-124,37	34,34
Quinidine hydrochloride monohydrate	Antiarrhythmic antimalarial	-124,42	24,25
Diethylcarbamazine citrate	Antihelmintic	-124,57	37,81
Alprostadil	Erectile Dysfunction treatment Vasodilator	-124,66	39,34
Methyldopate hydrochloride	Antihypertensive	-124,75	24,35
Ozagrel hydrochloride	Antianginal	-124,78	31,72
Progesterone	Progestogen	-125,25	35,51
Epiandrosterone	Anabolic	-125,94	39,83
Camylofine chlorhydrate		-126,38	35,61
Aminohippuric acid		-126,73	27,79
Spiperone	Antipsychotic	54,85	69,33
Nifurtimox		-127	41,36
Triflupromazine hydrochloride	Antiemetic antipsychotic anxiolytic	-127,23	39,11
Melatonin	Anticonvulsant antioxidant immunostimulant	-127,29	42,79
Omeprazole	Antiulcer	-127,53	31,84
Zotepine	Antipsychotic	-128,12	30,6
Nizatidine	Antiulcer	-128,13	42,05
Budesonide	Anti-inflammatory	-128,25	34,65
(R) -Naproxen sodium salt	Anti-inflammatory	-128,4	23,73
(+,-)-Octopamine hydrochloride		-129,2	38,1
Artemisinin	Antimalarial	-129,28	32,52
Alfadolone acetate	Anesthetic	-129,58	40,79
Prilocaine hydrochloride	Local anesthetic	-129,98	43,77
Moricizine hydrochloride	Antiarrhythmic	-130,23	40,5
Lincomycin hydrochloride	Antibacterial	193,73	91,33
Sulpiride	Antidepressant antiemetic antipsychotic	-130,36	35,25
Clofibrate	Antilipemic	-130,53	46,06
Fluconazole	Antifungal	-130,61	37,78
Ribavirin	Antiviral	-130,84	35,17
Oxytetracycline dihydrate	Antibacterial	-16,96	62,92
Bendroflumethiazide	Antihypertensive diuretic	-130,92	40,66

Name	Therapeutic effect	% GraRS inhibition	% Growth inhibition
Bromocryptine mesylate	Antiparkinsonian	-130,94	45,21
Dibenzepine hydrochloride	Antidepressant	-131,21	25,23
Chlorothiazide	Antihypertensive diuretic	-131,94	30,24
Methoxamine hydrochloride	Antihypotensive vasoconstrictor	-132,01	39,04
Tinidazole	Antiamebic Antibacterial	-132,05	30,76
Corticosterone	Anti-inflammatory immunosuppressant	-132,28	41,04
Minocycline hydrochloride	Antibacterial	127,99	90,93
Adrenosterone		-132,47	31,12
Mifepristone	Abortifacient	-132,58	26,3
Loteprednol etabonate	Anti-inflammatory	-132,82	42,24
Candesartan	Antihypertensive	-132,93	39,9
Mephenytoin	Anticonvulsant	-133,02	40,9
Piperacillin sodium salt	Antibacterial	-133,23	48,29
Chlormezanone	Anxiolytic Muscle relaxant	-133,27	38,18
Fendiline hydrochloride	Antianginal	-133,84	26,42
Ondansetron Hydrochloride	Antianemic	-134,08	38,4
Piribedil hydrochloride	Antiparkinsonian Vasodilator	-134,34	34,7
Primidone	Anticonvulsant	-134,9	42,18
Cefoperazone dihydrate	Antibacterial	415,31	83,3
Memantine Hydrochloride	Anti-Alzheimer Antiparkinsonian Antispastic	-135	35,15
Carbidopa	Antiparkinsonian	-135,14	40,14
Cyclizine hydrochloride	Antiemetic antihistaminic antivertigo	-135,37	32,87
Alfaxalone	Anesthetic	-135,65	34,76
Lopinavir	Antiviral	-135,79	24,13
Aztreonam	Antibacterial	-135,83	48,79
Ethamsylate	Antiplatelet Hemostatic	-136,4	44,76
Etofylline	Antispastic Bronchodilator Cardiotonic	-136,54	38,06
Phentermine hydrochloride		-136,67	36,62
Hycanthone	Antihelmintic Antiparasitic	-136,75	40,49
Tibolone		-137	48,6
Altretamine	Antineoplastic	-137,25	40,74
Triflusal	Anticoagulant antiplatelet	-137,66	38,19
Anethole-trithione	Choleretic	-137,71	42,82
Mefloquine hydrochloride	Antimalarial	-138,02	49,41
Meloxicam	Anti-inflammatory	-138,12	40,13
Bretylium tosylate	Anesthetic Antiarrhythmic Antihypertenisve	-138,12	44,37
Thiorphan	Antidiarrheal	-138,31	29,8
1,8-Dihydroxyanthraquinone	Laxative Antiemetic	-52,31	60,06
Thioperamide maleate	Antiemetic	-138,85	38,19
Aripiprazole	Antipsychotic	-138,9	43,34
Amoxapine	Antidepressant antipsychotic	-139,29	48,46
Pilocarpine nitrate	Antiglaucoma	-139,73	44,71

Name	Therapeutic effect	% GraRS inhibition	% Growth inhibition
Dextromethorphan hydrobromide monohydrate	antitussive	-139,74	36,97
Mesoridazine besylate	Antipsychotic	-141,03	36,99
Acefylline	CNS stimulant	-141,37	23,22
Sulfamethizole	Antibacterial	-141,44	34,52
Vigabatrin hydrochloride	Anticonvulsant antiepileptic	-142,42	29,39
Propofol	Anesthetic Sedative	-142,44	43,98
Dihydroergotamine tartrate	Antimigraine	-142,53	42,19
Rimantadine Hydrochloride	Antiviral	-143,38	34,95
Lidoflazine	Antianginal Antiarrhythmic Vasodilator	-143,66	34,88
Alfacalcidol	Antiosteoporosis	-143,72	39,73
Sulfinpyrazone	Antiplatelet uricosuric	-143,9	28,41
Bromhexine hydrochloride	Expectorant	-144,22	39,94
Sulfaquinoxaline sodium salt	Antibacterial	-144,43	39,65
Dinoprost trometamol	Oxytocic	-144,43	36,91
Misoprostol	Antiulcer	-144,65	50,57
Dimaprit dihydrochloride		-144,8	41,03
Mebeverine hydrochloride	Antispastic	-144,87	28,48
Pirlindole mesylate	Antidepressant	-146,43	38,55
Zomepirac sodium salt	Anti-inflammatory	-147,45	33,24
Rimexolone	Anti-inflammatory	-147,58	40,8
Erlotinib	Antineoplastic	-147,91	35,07
Asenapine maleate	Antipsychotic	-148,15	39,29
Metaproterenol sulfate, orciprenaline sulfate	Bronchodilator	-148,44	33,5
Niridazole	Antihelmintic antiparasitic antiprotozoal	-148,46	43,94
Iohexol	Contrastant	-148,59	34,99
Clofazimine	Antibacterial	273,41	81,3
Tolvaptan	Antihypertensive diuretic	-148,71	36,42
Mephenesin	Anticonvulsant local anesthetic muscle relaxant	-149,28	49,28
Fursultiamine Hydrochloride	Anti-Alzheimer	-149,3	45,09
Fusidic acid sodium salt	Antibacterial	361,06	91,91
Benzathine benzylpenicillin	Antibacterial	-149,39	51,81
Dexrazoxane hydrochloride	Chemoprotectant	-149,51	39,66
Methylergometrine maleate	Hemostatic Oxytocic	-150,28	37,45
Risperidone	Antipsychotic	-151,26	41,65
Lofexidine	Antihypertensive	-151,52	36,39
Gabexate mesilate	Anticoagulant	-151,66	36,7
Nitrocaramiphen hydrochloride		-151,96	41,55
Dequalinium dichloride	Antibacterial Antiseptic	-131,97	78,57
Pentoxifylline	Bronchodilator Vasodilator	-153,1	34,83
Thalidomide	Hypnotic Immunosuppressant	-153,59	40,23

Name	Therapeutic effect	% GraRS inhibition	% Growth inhibition
Exemestane	Antineoplastic	-155,08	42,38
Letrozole	Antineoplatic	-155,32	40,66
Oxybutynin chloride	Antispastic	-155,46	32,53
Kanamycin A sulfate	Antibacterial	698,91	74,53
Amikacin hydrate	Antibacterial	1163,39	92,93
Atovaquone	Antimalarial antiprotozoal	-155,49	36,03
Butylscopolammonium (n-) bromide	Antispastic	-155,52	38,85
Metoprolol-(+,-) (+)-tartrate salt	Antiarrhythmic antihypertensive	-156,17	40,62
Flunarizine dihydrochloride	Anticonvulsant vasodilator	-156,22	31,21
Cortisol acetate	Anti-inflammatory	-156,38	32,87
Metaraminol bitartrate	Antihypotensive vasoconstrictor	-156,42	50,72
Oxiconazole Nitrate	Antifungal	-156,94	39,13
Tenoxicam	Analgesic anti-inflammatory antipyretic	-157,27	37,85
Glimepiride	Antidiabetic	-157,36	37,38
Tigecycline		-157,71	30,04
Amifostine		-157,96	42,07
Pizotifen malate	Antihistaminic Antimigraine Sedative	-158,08	40,75
Metixene hydrochloride	Antiparkinsonian antispastic	-158,81	27,38
Nifekalant	Antiarrhythmic	-159,2	42,17
Khellin	Antispastic antitussive vasodilator	-159,36	48,51
Dipivefrin hydrochloride	Antiglaucoma	-159,69	31,15
Pargyline hydrochloride	Antidepressant antihypertensive	-160,04	47,24
Caffeine	CNS Stimulant	-160,13	43,44
Paliperidone	Antipsychotic	-160,21	44,17
Brinzolamide	Antiglaucoma Diuretic	-160,6	40,06
Chlorprothixene hydrochloride	Antiemetic Antipsychotic	-160,78	38,82
Etofenamate	Anti-inflammatory	-161,47	48,49
Pimozide	Antipsychotic	-36,39	66,56
Pronethalol hydrochloride	Antianginal Antiarrhythmic Antihypertenisve	-161,74	36,26
Moclobemide	Antidepressant	-161,82	39,05
Dantrolene sodium salt	Muscle relaxant	-162,15	31,34
Fluorometholone	Anti-inflammatory	-162,37	27,17
Vinpocetine	CNS Stimulant Neuroprotectant Vasodilator	-35,51	67,13
Lisinopril	Antihypertensive vasodilator	-162,45	32,05
Calcipotriene	Antipsoriatic	-162,61	54,39
Fomepizole		-162,62	38,87
Probucol	Antilipermic Hypocholesterolemic	-163,46	37,5
Zuclopenthixol dihydrochloride	Antipsychotic Antiviral Sedative	-163,49	42,58
Zardaverine	Bronchodilator	-163,57	40,74
Levonordefrin	Vasoconstrictor	-164,58	35,06
Pefloxacine	Antibacterial	40,67	68,21
Praziquantel	Antihelmintic	-165,02	25,44

Name	Therapeutic effect	% GraRS inhibition	% Growth inhibition
Torsemide	Antihypertensive Diuretic	-165,09	44,22
Granisetron	Antiemetic	-166,09	52,05
Maprotiline hydrochloride	Antidepressant Anxiolytic	-166,35	41,55
Loratadine	Antihistaminic	-167,11	36,6
Azathioprine	Antineoplastic immunosuppressant	-167,21	50,85
Cyanocobalamin	Analgesic	-167,36	45,44
Flecainide acetate	Antiarrhythmic	-168,09	38,99
Dicloxacillin sodium salt hydrate	Antibacterial	119,67	81,21
Famciclovir	Antiviral	-168,18	48,74
Miglitol	Antidiabetic	-168,28	40,92
Deptropine citrate	Antihistaminic Bronchodilator Vasodilator	-168,93	49,42
Cyclosporin A	Immunosuppressant	-169,41	39,86
Antazoline hydrochloride	Antihistaminic sedative	-31,51	68,68
5-fluorouracil	Antineoplastic	-31,36	70,27
Tracazolate hydrochloride	Anticonvulsant Sedative	-169,77	39,01
Cinoxacin	Antibacterial	-170,3	39,83
Nebivolol hydrochloride	Antihypertensive	-170,57	47,84
Medrysone	Anti-inflammatory	-170,79	41,82
Ibandronate sodium	Antiosteoporosis	-171,09	36,43
Bupivacaine hydrochloride	Local anesthetic	-171,16	36,61
Carbarsone	Antiamebicantiprotozoal	-171,23	41,43
Linezolid	Antibacterial	93,91	62,09
Fenoterol hydrobromide	Bronchodilator tocolytic	-171,75	36,57
Meclocycline sulfosalicylate	Antibacterial	87,48	77,44
Chlorthalidone	Antihypertensive Diuretic	-172,61	39,4
Melengestrol acetate		-173	46,59
Pipenzolate bromide	Antispastic	-173,25	41,84
Ceforanide	Antibacterial	-20,07	69,45
Oxcarbazepine	Anticonvulsant	-173,28	44,74
Cefixime	Antibacterial	-63,52	67,49
Pirenperone		-173,75	38,83
Mebhydroline 1,5-naphtalenedisulfonate	Antihistaminic	-173,84	47,01
Tosufloxacin hydrochloride	Antibacterial	-59,88	78,57
Methylprednisolone, 6-alpha	Anti-inflammatory Immunosuppressant	-173,92	38,12
Rifapentine	Antibacterial	101,55	78,07
Zimelidine dihydrochloride monohydrate	Antidepressant	-28,25	55,63
Mometasone furoate	Anti-inflammatory	-174,08	38,79
Closantel	Antihelmintic antiparasitic	141,43	75,63
Bisacodyl	Laxative	-174,76	38,82
Tegafur	Antineoplastic	-175,65	46,24
Trimetozine	Sedative	-176,09	37,52

Name	Therapeutic effect	% GraRS inhibition	% Growth inhibition
Streptozotocin	Antineoplastic	-176,31	40,9
Glycopyrrolate	Antispastic	-176,37	41,35
Triprolidine hydrochloride	Antihistaminic sedative	-177,47	29,17
Cinnarizine	Antihistaminic antivertigo sedative	-26,35	69,62
Indomethacin	Analgesic anti-inflammatory antipyretic	-177,56	35,46
Zaleplon	Hypnotic sedative	-178,45	46,15
Isosorbide dinitrate	Antianginal	-178,87	26,98
Halcinonide	Anti-inflammatory antipruritic	-179,07	35,06
Cefotiam hydrochloride	Antibacterial	-1007,95	95,38
Betahistine mesylate	Vasodilator	-179,43	36,15
Azelastine hydrochloride	Antihistaminic	-180,09	41,22
Ribostamycin sulfate salt	Antibacterial	-180,56	47,77
Dobutamine hydrochloride	Analeptic Cardiotonic Positive inotropic	-180,61	38,91
Folinic acid calcium salt	Antianemic	-180,72	40,09
Bosentan	Vasodilator	-180,86	53,39
Diperodon hydrochloride	Local anesthetic	-180,87	34,04
Alendronate sodium	Antiosteoporosis	-180,91	29,58
Ibutilide fumarate	Antiarrhythmic	-181,04	27,87
Acetylcysteine	Mucolytic	-181,64	45,19
Levetiracetam	Anticonvulsant	-182,71	24,87
Desloratadine	Antihistaminic	-22,83	62
Cetirizine dihydrochloride	Antihistaminic antipruritic	-182,79	37,78
Amlodipine	Antihypertensive	-183,03	40,87
Vancomycin hydrochloride	Antibacterial	-820,38	95,15
Clofibric acid	Antilipemic	-183,34	42,53
Finasteride	Anti-alopecia antineoplastic	-184,05	32,97
Phenacetin	Analgesic antipyretic	-185,28	39,03
Cisapride	Gastroprokinetic	-185,94	40,33
Betamethasone	Anti-inflammatory Antipruritic Immunosuppressant	-186,53	39,25
Clorgyline hydrochloride	Antidepressant	-187,19	43,51
Dacarbazine	Antineoplastic	-187,32	45,14
Metrizamide	Contrastant	-188,45	44,17
Zaprinast	Erectil dysfunction treatment	-188,96	39,8
Lacidipine	Antihypertensive	-188,97	43,85
Quetiapine hemifumarate	Antipsychotic	-189,06	54,02
Imipenem	Antibacterial	-1799,83	97,05
Methiothepin maleate	Antipsychotic	-189,16	48,36
Pramoxine hydrochloride	Local anesthetic	-189,79	40,71
Biotin		-189,81	40,82
Clenbuterol hydrochloride	Antiasthmatic Bronchodilator Tocolytic	-189,88	44,23
Diphenidol hydrochloride	Antiemetic antivertigo	-189,94	30,46

Name	Therapeutic effect	% GraRS inhibition	% Growth inhibition
Chlorotrianisene	Antineoplastic	-190	43,91
Tetramisole hydrochloride	Antihelmintic antiparasitic immunomodulator	-190,8	37,06
Ropinirole hydrochloride	Antiparkinsonian	-190,86	42,74
Dronedarone hydrochloride	Antiarrhythmic	-191	41,63
Galanthamine hydrobromide	Analgesic anti-alzheimer anti-fatigue	-191,3	44,44
Mesna	Chemoprotectant	-191,31	47,78
Roxatidine Acetate hydrochloride	Antiulcer	-191,43	43,96
Thiostrepton	Antibacterial	10,1	91,31
Nitrofural	Antibacterial	-191,59	38,05
Fenofibrate	Hypocholesterolemic Lipid-lowering Uricosuric	-191,6	35,52
Rifampicin	Antibacterial	-360,18	92,86
THIP Hydrochloride	sedative	-191,79	38,76
Clomipramine hydrochloride	Antidepressant	-191,8	32,4
Tropisetron hydrochloride	Antiemetic	-192,25	36,97
(S)-(-)-Atenolol	Antianginal antiarrhythmic antihypertensive	-192,32	39,47
Thiethylperazine dimalate	Antiemetic Antivertigo	-192,36	50,1
Flumethasone	Anti-inflammatory	-192,38	45,47
Grepafloxacin		82,7	82,91
Furosemide	Antihypertensive Diuretic	-192,43	35,68
D,L-Penicillamine	Analgesic	-193,14	31,31
Fenoprofen calcium salt dihydrate	Anti-inflammatory	-193,33	41,23
Clemastine fumarate	Antiemetic antihistaminic sedative	-193,36	38,14
Zidovudine, AZT	Antiviral	-193,49	36,62
Vidarabine	Antiviral	-193,53	46,46
Cefotetan	Antibacterial	-194,65	49,78
Pimethixene maleate	Antihistaminci antitussive Bronchodilator	-195,39	29,95
Carisoprodol	Analgesic antipyretic muscle relaxant	-195,66	47,09
Probenecid	Antigout uricosuric	-195,72	35,28
Benzocaine	Local anesthetic	-195,88	15,71
Tobramycin	Antibacterial	-485,01	87,09
Isoetharine mesylate salt	Bronchodilator	-196,04	36,65
Tiapride hydrochloride	Antiemetic antipsychotic anxiolytic	-196,81	40,91
Pemirolast potassium	Anti-inflammatory Antipruritic Antihistaminic	-197,24	41,37
Isopropamide iodide	Antiulcer	-197,74	43,01
Amprenavir	Antiviral	-198,04	40,69
Pyrilamine maleate	Antihistaminic antipruritic antitussive	-198,32	40,25
Bacampicillin hydrochloride	Antibacterial	-199,63	43,09
Fenipentol	Choleretic	-200,02	42,45
Acamprosate calcium		-200,52	47,87
Methapyrilene hydrochloride	Antihistaminic Sedative	-201,74	36,89
Sisomicin sulfate	Antibacterial	-8,89	57,15

Name	Therapeutic effect	% GraRS inhibition	% Growth inhibition
Ipsapirone		-202,19	49,37
Tenatoprazole	Antiulcer	-202,4	40,53
Carmofur	Antineoplastic	-7,88	58,75
Cefpiramide	Antibacterial	-7,51	60,64
Topotecan	Antineoplastic	-203,08	53,57
Sulfabenzamide	Antibacterial	-203,71	29,17
Voriconazole	Antifungal	-203,78	47,27
Cefsulodin sodium salt	Antibacterial	-5,82	65,88
Nisoldipine	Antianginal antihypertensive	-205,35	35,05
Metergoline	Antiprolactin	-206,31	46,57
Pregnenolone	Anabolic anti-inflammatory	-207,03	39,31
Suloctidil	Antiplatelet vasodilator	-207,41	41,52
Alizapride hydrochloride	Antiemetic	-207,57	44,25
Clopamide	Antihypertensive Diuretic	-207,72	47,01
Leflunomide	Immunosuppressant	-208,18	32,32
Thioguanosine	Antineoplastic	-208,21	41,13
Methacholine chloride		-208,69	42,28
Aniracetam	Anti-alzheimer	-209,7	48,9
Cortisone	Anti-inflammatory Immunosuppressant	-210,96	40,33
Beta-Escin	Antineoplastic diuretic	-211,18	42,73
Butenafine Hydrochloride	Antifungal	-211,75	53,9
Norethindrone	Contraceptive	-212,05	39,33
Sulfachloropyridazine	Antibacterial	-212,2	34,6
Androsterone	Anabolic	-212,35	37,25
Acetopromazine maleate salt	Antiemetic antipsychotic antitussive	-212,97	41,38
Bambuterol hydrochloride	Bronchodilator Tocolytic	-213,37	36,56
Prednisolone	Anti-inflammatory Immunosuppressant	-214,55	40,16
Bimatoprost	Antiglaucoma	-214,63	40,86
Practolol	Antianginal Antihypertensive	-215,33	35,43
Dyclonine hydrochloride	Local anesthetic	-215,84	37,99
Etretinate	Antipsoriatic	-217,14	46,43
Buflomedil hydrochloride	Vasodilator	-217,14	43,52
Guaifenesin	Bronchodilator expectorant	-218,14	46,77
Amoxicillin	Antibacterial	-218,73	48,36
Ethisterone	Contraceptive	-218,88	35,33
Glafenine hydrochloride	Analgesic	-219,16	38,27
Efavirenz	Antiviral	-219,95	48,65
Fludrocortisone acetate	Anti-inflammatory antipruritic	-220,48	37,57
Argatroban	Anticoagulant	-220,79	44,69
Pitavastatin calcium	Hypocholesterolemic	-221,75	47,2
Hydroxychloroquine sulfate	Antimalarial	-222,05	48,01
Papaverine hydrochloride	Antispastic antitussive erectile dysfunction	-222,84	46,71

Name	Therapeutic effect	% GraRS inhibition	% Growth inhibition
	treatment		
Proadifen hydrochloride	Local anesthetic	-224,52	45,55
Spiramycin	Antibacterial	6,02	65,82
Escitalopram oxalate	Antidepressant	-224,95	39,74
Benzamil hydrochloride	Antihypertensive diuretic	-225,18	35,27
Estradiol-17 beta	Antigonadotropin	-225,5	45,32
Zafirlukast	Antiasthmatic	97,7	76,25
Zopiclone	Hypnotic sedative	-225,91	47,08
Pranlukast	Antiasthmatic	-227,03	38,21
Clonidine hydrochloride	Analgesic antihypotensive sedative	7,96	58,79
Labetalol hydrochloride	Antihypotensive	-227,54	39,22
Hydrocortisone base	Anti-inflammatory	-228,11	42,38
Guanadrel sulfate	Antihypertenisve	-228,59	45,51
Dydrogesterone	Progestogen	-228,84	45,82
Cycloheximide	Antibacterial	-230,46	42,63
Isoniazid	Antibacterial	-231,26	25,29
Tirofiban hydrochloride	Antiplatelet	-231,86	49,29
Oxibendazol		-233,13	52,06
Mizolastine		-235,15	47,3
Tetracaïne hydrochloride		-235,34	39,39
Picrotoxinin	Analeptic	-237	40,42
Dimenhydrinate	Antiemetic antihistaminic antivertigo	-239,52	40,87
Terazosin hydrochloride	Antihypertensive	-239,59	44,48
Vincamine	CNS Stimulant Vasodilator	-239,9	34,95
Meclofenoxate hydrochloride	CNS Stimulant	-239,98	45,96
Xylazine	Analgesic sedative	-240,09	54,4
Dexamethasone acetate	Anti-inflammatory immunosuppressant	-240,22	42,34
Doxepin hydrochloride	Anticonvulsant antidepressant antipruritic	-240,65	37,76
Disopyramide	Antiarrhythmic	-240,7	34,42
Doxorubicin hydrochloride	Antibacterial antineoplastic immunosuppressant	14,31	61,8
Tizanidine hydrochloride	Muscle relaxant	-241,2	51,82
Butamben	Anesthetic	-241,28	48,68
Sulfamerazine	Antibacterial	-241,28	39,67
Podophyllotoxin	Antiviral	-241,42	47,82
Bumetanide	Diuretic	-241,45	36,21
Trioxsalen		-242,77	42,13
Florfenicol	Antibacterial	15,77	66,02
Fipexide hydrochloride	Anti-fatigue CNS stimulant	16,02	62,49
Digoxin	Cardiotonic	-243,64	47,02
Sulfamethazine sodium salt	Antibacterial	-243,86	46,28
Methimazole		-244,16	48,79

Name	Therapeutic effect	% GraRS inhibition	% Growth inhibition
Norgestrel-(-)-D	Contraceptive	-244,58	48,39
Acemetacin	Anti-inflammatory	-245,1	39,07
Pinaverium bromide	Antipastic	-745,12	93,96
Nortriptyline hydrochloride	Antidepressant CNS stimulant	-245,81	36,4
Benfotiamine		-246,03	40,17
Benazepril hydrochloride	Antihypertensive	-246,13	52,78
Diosmin		-247,64	54,83
Hexylcaine hydrochloride	Anesthetic	-248,37	45,8
Temozolomide	Antineoplastic	-249,74	48,89
Celiprolol hydrochloride	Antianginal antihypertensive	-249,89	51,28
Dimethisoquin hydrochloride	Antipruritic lical anesthetic	-252,01	53,39
Drofenine hydrochloride	Antispastic	-252,06	43,32
Emedastine	Antihistaminic	-252,44	51,39
Sulfameter	Antibacterial	-253,12	43,16
Desipramine hydrochloride	Antidepressant CNS Stimulant	-253,36	45,93
Ketotifen fumarate	Antihistaminic	-254,34	35,52
Acipimox	Antilipemic	-256,89	53,12
Colchicine	Antigout Anti-inflammatory	-257,58	44,38
Folic acid		-259,46	51,71
Citalopram Hydrobromide	Antidepressant	-260,3	35,94
Pranoprofen	Anti-inflammatory	-260,41	47,6
Demecarium bromide	Antiglaucoma	-265,08	53,76
Pyridoxine hydrochloride		-266,05	53,65
Thioproperazine dimesylate	Antiemetic antipsychotic	27,55	56,28
Lanatoside C	Cardiotonic	-269,8	39,07
Mercaptopurine	Immunosuppressant	-272,06	53,63
Reboxetine mesylate	Antidepressant	-272,43	50,71
Mepenzolate bromide	Antispastic antiulcer	-274,45	40,21
Nalidixic acid sodium salt	Antibacterial	-274,99	41,26
Suxibuzone	Analgesic anti-inflammatory antipyretic	-275,31	40,27
Racecadotril	Antidiarrheal	-275,67	54,96
Dirithromycin	Antibacterial	32,47	69,13
Triamcinolone	Anti-inflammatory immunosuppressant	-276,79	43,59
Venlafaxine	Antidepressant	-278,74	49,73
Phenazopyridine hydrochloride	Analgesic	-279,32	49,57
Estropipate		-286,23	39,89
Tetraethylenepentamine pentahydrochloride	Antilipemic	-290,81	41,19
Oxacillin sodium	Antibacterial	-2146,59	96,8
Tranilast	Antiallergic	-291,5	52,04
Trihexyphenidyl-D,L Hydrochloride	Antiparkinsonian	-298,84	42,47
Alclometasone dipropionate	Anti-inflammatory	-305,4	45,83

Name	Therapeutic effect	% GraRS inhibition	% Growth inhibition
Dipyrone	Analgesic antiasthmatic antipyretic	-306,55	33,48
Homochlorcyclizine dihydrochloride	Antihistaminic sedative	-306,56	39,22
Benzethonium chloride	Antibacterial antiseptic	-316,26	49,84
Mirabegron		-320,08	45,79
Sumatriptan succinate	Antimigraine	-323,76	46,63
Doxofylline	Bronchodilator	-325,87	46,56
Perhexiline maleate	Antianginal	-326,3	39,93
Sulfapyridine	Antibacterial	-330,79	48,84
Tetrahydrozoline hydrochloride	Nasal decongestant vasoconstrictor	-337,86	44,24
Piroxicam	Analgesic anticoagulant anti-inflammatory	-337,96	36,5
Zileuton	Antiasthmatic	-341,85	44,19
Cefmetazole sodium salt	Antibacterial	-2413,38	97,12
Tylosin	Antibacterial	45,13	85,17
Quinacrine dihydrochloride hydrate	Antihelmintic antileishmanial antimalarial	59,1	62,81
Thiamine hydrochloride	Immunostimulant	-346,43	43,69
Bromopride	Antiemetic	-349,22	49,43
Ethotoin	Anticonvulsant	-350,77	50,03
Warfarin	Anticoagulant	-352,58	45,34
Famprofazone	Analgesic antipyretic	-353	46,62
3-alpha-Hydroxy-5-beta-androstan-17-one		-353,82	40,87
Irsogladine maleate	Antiulcer	-354,28	40,84
Opipramol dihydrochloride	Antidepressant antipsychotic	-355,1	44,38
Ivermectin	Antihelmintic antiparasitic	81,94	68,9
Epirubicin hydrochloride	Antineoplastic	83,01	60,93
Succinylsulfathiazole	Antibacterial	-362,6	46,99
Pempidine	Antihypotenisve vasodilator	-371,17	46,07
Methyl benzethonium chloride	Antibacterial	-1322,89	96,66
Irinotecan hydrochloride trihydrate	Antineoplastic	-387,09	42,15
Midodrine hydrochloride	Antihypertensive	-392,31	37,84
Tramadol hydrochloride	Analgesic	-410,34	49,33
Cephalothin sodium salt	Antibacterial	-972,09	94,62
Cefuroxime sodium salt	Antibacterial	-837,14	87,29
Ranitidine hydrochloride	Antiulcer	-413,31	35,38
Ampyrone	Analgesic anticoagulant anti-inflammatory	-418,54	39,74
Secnidazole	Antiamebic	-418,93	48,42
Benzbromarone	Antianginal Antigout Antispastic	131,47	56,37
Tranexamic acid	Hemostatic	-428,99	37,53
Chlorcyclizine hydrochloride	Antiemetic antihistaminic sedative	-434,53	54,63
Diphenylpyraline hydrochloride	Antihistaminic antipruritic sedative	-488,99	50,6
Troleandomycin	Antibacterial	289,19	76,05
